# The plasticity of the grapevine berry transcriptome

**DOI:** 10.1186/gb-2013-14-6-r54

**Published:** 2013-06-07

**Authors:** Silvia Dal Santo, Giovanni Battista Tornielli, Sara Zenoni, Marianna Fasoli, Lorenzo Farina, Andrea Anesi, Flavia Guzzo, Massimo Delledonne, Mario Pezzotti

**Affiliations:** 1 Department of Biotechnology, University of Verona, Strada Le Grazie 15 - Ca' Vignal, 37134 Verona, Italy; 2 Department of Computer, Control, and Management Engineering Antonio Ruberti, Sapienza University of Rome, Via Ariosto 25, 00185 Rome, Italy

**Keywords:** Phenotypic plasticity, Transcriptome, Grapevine

## Abstract

**Background:**

Phenotypic plasticity refers to the range of phenotypes a single genotype can express as a function of its environment. These phenotypic variations are attributable to the effect of the environment on the expression and function of genes influencing plastic traits. We investigated phenotypic plasticity in grapevine by comparing the berry transcriptome in a single clone of the vegetatively-propagated common grapevine species *Vitis vinifera *cultivar Corvina through 3 consecutive growth years cultivated in 11 different vineyards in the Verona area of Italy.

**Results:**

Most of the berry transcriptome clustered by year of growth rather than common environmental conditions or viticulture practices, and transcripts related to secondary metabolism showed high sensitivity towards different climates, as confirmed also by metabolomic data obtained from the same samples. When analyzed in 11 vineyards during 1 growth year, the environmentally-sensitive berry transcriptome comprised 5% of protein-coding genes and 18% of the transcripts modulated during berry development. Plastic genes were particularly enriched in ontology categories such as transcription factors, translation, transport, and secondary metabolism. Specific plastic transcripts were associated with groups of vineyards sharing common viticulture practices or environmental conditions, and plastic transcriptome reprogramming was more intense in the year characterized by extreme weather conditions. We also identified a set of genes that lacked plasticity, showing either constitutive expression or similar modulation in all berries.

**Conclusions:**

Our data reveal candidate genes potentially responsible for the phenotypic plasticity of grapevine and provide the first step towards the characterization of grapevine transcriptome plasticity under different agricultural systems.

## Background

Most organisms show evidence of phenotypic plasticity, that is, the ability of a single genotype to produce a range of phenotypes as a function of its environment [1]. This represents a key strategy to maximize fitness when challenged by environmental heterogeneity [2]. Moreover, sessile organisms such as plants rely on phenotypic plasticity to cope with the changing environment, so the phenomenon has a significant impact on evolution, ecology and agriculture [3-5] as well as on plant responses and adaption in the context of rapid climate change [3]. Although phenotypic plasticity is an important ecological phenomenon, the underlying genetic and molecular mechanisms remain still poorly characterized [6].

Phenotypic variation between species and organism of the same species may reflect differences in gene structure as well as differences in gene expression, but phenotypic plasticity among clones of the same genotype is likely to be much more dependent on differential gene expression in different environments [7]. The availability of high-throughput expression profiling technologies now makes it possible to analyze gene expression (activity and spatiotemporal characteristics) on a global scale, so that transcriptome plasticity can be investigated directly [7-9]. Transcriptome plasticity has recently been described in model organisms such as the fruit fly *Drosophila melanogaster *[10], the mouse *Mus musculus *[11], and the nematode *Caenorhabditis elegans *[12]. Other studies have considered the transcriptional basis of phenotypic variation in non-model organisms in the wild or under controlled environments [13-16].

Few comprehensive studies have been reported for plants cultivated in open fields, where they are exposed to multiple environmental stimuli that induce complex responses in terms of gene expression, metabolic activity, and epigenetic modifications. These studies have focused mainly on transcriptome remodeling in response to individual abiotic factors [17,18] or during a single developmental process [19]. Recently Richards *et al. *[20] analyzed the genome-wide gene expression pattern in two accessions of *Arabidopsis thaliana *and explored the correlation between gene expression and natural environmental fluctuations. This revealed that accession is an important component of transcriptional variation among individuals in the field.

Grapevine (*Vitis *spp., family *Vitaceae*) is the most widely-cultivated perennial fruit crop in the world, with 67.5 million tons of berries produced in 2011 [21]. The berries are characterized by considerable phenotypic plasticity, with the same clone showing variability within individual berries, among berries within a cluster, between clusters on a vine, and among vines in the vineyard, according to both environmental factors and viticulture practices [22]. This can be considered a burden because the berries may mature unevenly and display large interseasonal fluctuations in quality, but it also offers advantages such as the ability to adapt existing cultivars to specific growing regions and to produce different wines from the same cultivar [23].

We investigated the extent to which phenotypic plasticity in grape berries reflects underlying changes in the transcriptome by using NimbleGen microarray technology in combination with the complete grapevine genome sequence [24] to study global gene expression profiles in a single clone of *Vitis vinifera *cv Corvina cultivated in different vineyards and harvested at different developmental stages over 3 consecutive years. We monitored the transcriptomic response to seasonal changes, highlighting transcripts expressed under both normal and unusual weather conditions. We identified the component of the grapevine transcriptome that is plastic, allowing different developmental responses under diverse growing conditions. We studied the relationships among differential gene expression profiles, growing conditions and ripening parameters and identified several putative candidate genes for the definition of berry quality traits. The large-scale sampling procedure we used also allowed the identification of non-plastic genes such as constitutive housekeeping genes that provide useful references for quantitative expression analysis, and developmental markers that may be suitable for the on-field monitoring of berry ripening.

## Results

### Sampling strategy and seasonal climate analysis

**Figure 1:**
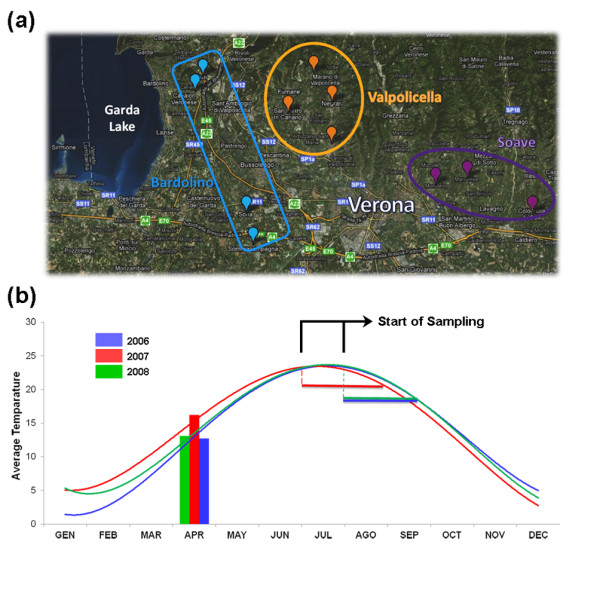
**Sampling macro-areas and temperature trends in the Verona region, Veneto, Italy**. **(a) **Sampling locations of *Vitis vinifera *cv Corvina clone 48 berries near Verona, Italy. We chose 11 different vineyards from the three most important wine production macro-areas of the region: Bardolino, Valpolicella, and Soave. **(b) **Average annual temperature trends. Temperature measurements were averaged from three recording stations located near each macro-area. The start and end point of sampling are indicated for each year.

*Vitis vinifera *cv Corvina clone 48 berries were harvested from different vineyards, each located in one of the three most important wine production macro-areas of the Verona region (Bardolino, Valpolicella, and Soave). The vineyards were selected on the basis of the site geographical coordinates to maximize differences in environmental conditions (altitude and soil type) and agricultural practices (training system, orientation of the rows, planting layout, vineyard age, and rootstock type) in each of the selected vineyards (Figure 1a; see Additional File 1, Table S1). Berry samples were harvested from all the vineyards on the same day and three biological replicates were taken at each of three different developmental stages (veraison - that is, the term used by viticulturists to indicate the onset of ripening -, mid-ripening, and fully-ripe). A complete list of all samples collected for this study is shown in Additional File 2, Table S2. In brief, sample names are composed by vineyard abbreviations (see Additional File 1, Table S1), followed by the indication of the harvesting year (06, 07, or 08), by the indication of the developmental stage (1, 2, or 3) and by the description of the biological replicate (A, B, or C). The berry ripening stage was verified by measuring three traditional ripening parameters (°Brix, total anthocyanin levels, and total acidity) as well as the ratio between quercetin-3-O-glucoside and quercetin-3-glucoronide, reflecting the fact that ripening Corvina berries progressively lose the former and accumulate the latter [25] (see Additional File 3, Table S3).

The same sampling procedure was repeated over 3 consecutive growth years (2006, 2007, and 2008). In order to obtain samples harvested at a similar phenological phase in the 3 years, the collecting times were advanced or delayed based on seasonal climate conditions and/or agro-meteorological trends. Daily temperature recording suggested that the 2007 season experienced a much warmer spring than the 2006 and 2008 ones (Figure 1b). In a comprehensive study of the relationship between grapevine phenology and climate change in the Veneto region over the period 1964 to 2009, the early spring of 2007 was noted for the highest average temperature (with near-normal precipitation) in the entire 45-year period. The 2007 veraison-to-harvest period was nearly 2 weeks ahead of time compared to the last decade average [26].

Based on the traditional and metabolic parameters discussed above, and with the appropriate inter-annual corrections taken into account, the collected samples were considered homogenously and uniformly ripe among different vineyards and growth years at each developmental stage (see Additional File 3, Table S3).

### The impact of season climate on the berry transcriptome

We used the NimbleGen 090918_Vitus_exp_HX12 microarray to investigate the Corvina berry transcriptome at three developmental stages harvested during the 2006-2008 period from four vineyards (AM, CS, MN, and PSP) chosen to maximize climatic and agricultural differences (see Additional File 1, Table S1 and Additional File 2, Table S2). The vineyards therefore represented all three of the macro-areas we considered (Bardolino, Valpolicella, and Soave) and a range of diverse environmental and agricultural parameters including three rootstock types, two altitudes, two vineyard training systems, and rows facing in different directions.

The 108-sample dataset (four vineyards, three developmental stages, three biological replicates, 3 years) was further dissected into three stage-specific 36-sample datasets (four vineyards, one developmental stage, three biological replicates, 3 years). We generated a Pearson's distance correlation matrix for each dataset to compare the transcriptome from each sample. These values were converted into distance coefficients to define the height of a dendrogram.

**Figure 2:**
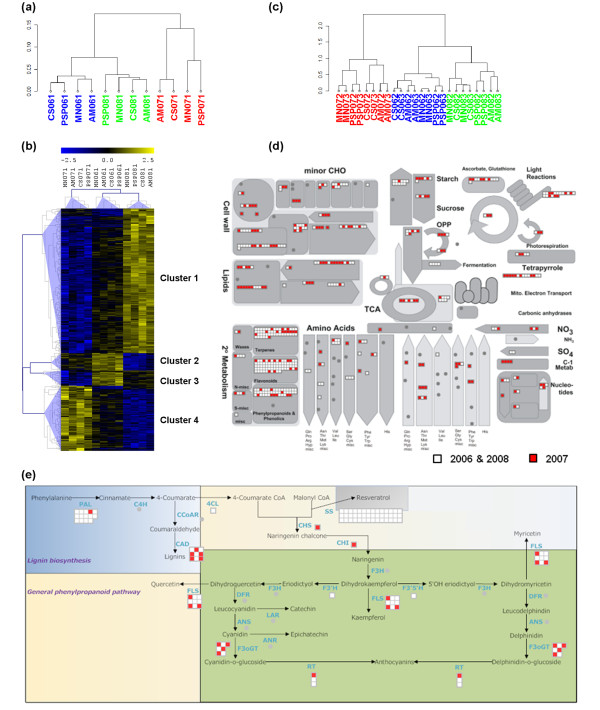
**Transcriptomic reprogramming in different climatic seasons**. **(a) **Cluster dendrogram of the first developmental stage dataset using the average expression value of the three biological replicates. Pearson's correlation values were converted into distance coefficients to define the height of the dendrogram. Sample names are composed by vineyard abbreviation followed by the indication of the harvesting year (06, 07, or 08) and by the indication of the developmental stage (1). Blue, green, and red indicate samples harvested in 2006, in 2008, and in 2007, respectively. Data are the average of the three biological replicates. **(b) **Hierarchical clustering analysis of transcripts that were differentially modulated among different seasons in first-stage samples. Kruskal-Wallis analysis of variance (*P *<0.01, three groups) was used to define transcripts whose expression is modulated in at least one growing season. Pearson's correlation distance was used as the metric to create the transcriptional profile dendrogram. Sample names are composed by vineyard abbreviation followed by the indication of the harvesting year (06, 07, or 08) and by the indication of the developmental stage (1). Data are the average of the three biological replicates. **(c) **Cluster dendrogram of the second and the third developmental stage datasets using the average expression value of the three biological replicates. Pearson's correlation values were converted into distance coefficients to define the height of the dendrogram. Sample names are composed by vineyard abbreviation followed by the indication of the harvesting year (06, 07, or 08) and by the indication of the developmental stages (2 or 3). Blue, green, and red indicate samples harvested in 2006, in 2008, and in 2007, respectively. Data are the average of the three biological replicates. MapMan software (v. 3.5) was used to visualize ripe berry genes specifically expressed in the 2006/2008 (white) and 2007 (red) growing seasons in an overview of metabolism **(d) **and focusing on the phenylpropanoid pathway **(e)**.

Berry samples collected at veraison clearly clustered in relation to the growth year and not in relation to the growing sites (Figure 2a). The 2006 and 2008 seasons correlated more closely than either did to the 2007 season, indicating that the high spring temperatures in 2007 had an impact on berry development. To gain insight into the physiological and molecular factors underlying this separation between samples, we carried out a three-group Kruskal-Wallis non-parametric analysis of variance (*P *<0.01) on the complete first-stage dataset. Hierarchical clustering (HCL) analysis on the resulting 625 genes, whose expression profiles showed a significant difference in modulation in at least 1 year, revealed four major groups (Figure 2b; see Additional File 4, Dataset S1).

Cluster 1 included 373 genes showing higher expression levels in 2008 compared to low levels in 2007. Most of these genes represented the 'DNA/RNA metabolic process' functional category, including several encoding histones, pentatricopeptide proteins, DNA replication proteins, mRNA cap guanidine methyltransferases, and RNA-binding proteins. The 'Transcription' functional category was also strongly represented, including genes encoding bHLH, MYB, bZIP2, and zinc finger transcription factors. The strong representation of these genes suggested a profound remodeling of the transcriptome between the growth years. We also identified stress response genes encoding two thaumatins, a metallothionein [27], and at least four senescence-associated proteins.

Cluster 2 contained 47 genes that were expressed at high levels in 2006 but at low levels in 2008. This included six genes related to hormone metabolism, four of which involved in the response to abscissic acid (ABA), which plays a pivotal role in development, adaptation to dehydration stress [28] and the production of reactive oxygen species (ROS). Given the presence of an early response to dehydration (ERD) protein and of two nudix hydrolases, which have recently been shown to maintain redox homeostasis [29], it is likely that the 2006 season was exposed to greater dehydration stress than the 2008 one.

Cluster 3 comprised 39 genes that were expressed at significantly higher levels in 2006 than 2007. These included genes encoding three expansin proteins directly involved in cell wall expansion [30], and a xyloglucan endotransglucosylase/hydrolase (XTH), which modifies hemicellulose during wall expansion and fruit softening and therefore suggests a direct influence of the growth year condition on cell wall metabolism [31]. Cluster 3 also included four genes related to carbohydrate synthesis, encoding sucrose synthase 2, a transketolase, a phosphomannomutase, and a galactokinase.

Finally, cluster 4 comprised 168 genes expressed at significantly higher levels in 2007 than in 2008. Interestingly, this group included genes encoding at least 10 disease-resistance proteins and heat shock factors. We also identified genes involved in the oxidative burst (two monooxygenases and a respiratory burst oxidative protein B) as well as two alcohol dehydrogenases involved in fermentative metabolism. The upregulation of these genes confirms that severe stress was imposed upon developing berries during the 2007 growing season.

Whereas the veraison berry dendrogram showed predominantly year-specific clustering, the ripening berry dendrograms were organized in a different manner (see Additional File 5, Figure S1a and S1b). Year-specific modulated genes in these samples were identified by normalizing the microarray fluorescence intensity values against the corresponding veraison values, resulting in a dendrogram showing samples clustered according to the growth year (Figure 2c). This indicated that the mid-ripening and late-ripening datasets could be also screened for year-specific modulated transcripts.

To explore the transcriptomic differences between the mid-ripening and late-ripening samples when comparing average climate growth year (2006/2008) and the 2007 season characterized by an exceptionally warm spring, we carried out a paired two-group t-test analysis which revealed 4,775 genes showing significant (*P *<0.01) differential transcription in one of the two groups (see Additional File 6, Dataset S2). After averaging the fluorescence intensity of all the samples within one group, we used MapMan [32] to visualize genes that were induced either specifically in the 2006/2008 seasons or specifically in 2007 (Figure 2d). We noted that enzymes involved in cell wall structural modifications (especially cellulose synthases, pectinesterases, and xyloglucan endotransglucosylase/hydrolases) were represented to a great extent in the 2006/2008 group, as previously observed in cluster 3 (Figure 2b), suggesting that the expression of these genes is affected by the different season climate. Genes with a role in amino acid metabolism were also induced in 2006/2008, indicating that the management of nitrogen-base substances is impaired under extreme temperatures. However, the major difference between the growth years involved secondary metabolism (Figure 2d), particularly the biosynthesis of phenylpropanoid derivatives in the 2006 and 2008 berries. This was indicated by the induction of genes encoding several phenylpropanoid-related enzymes (for example, phenylalanine ammonia lyase, PAL, and cinnamyl alcohol dehydrogenase (CAD), including a high number of stilbene synthases (STSs), controlling the key step for the synthesis of stilbene compounds (Figure 2e). LC-ESI-MS metabolomic analysis of the same samples used for RNA extraction confirmed that phenylpropanoid-derived compounds such as stilbenes, viniferins, hydroxycinnamic acids, and the flavonoid catechins and epicatechins were less abundant in the 2007 season compared to the 2006/2008 seasons, strongly supporting the transcriptomic data (see Additional File 7, Figure S2). This suggests that the profound reprogramming of the berry transcriptome under diverse meteorological conditions includes metabolic pathways contributing to ripe berry qualitative traits, thus influencing the commercial value of the grapes.

### Adaptation of the berry transcriptome to different environments and growing conditions

We focused on the impact of different environments and growing conditions by analyzing berries from the 2008 season, which appeared to be less influenced by the climate than the other growth years (see Additional File 8, Figure S3). We extended the analysis to include all 11 vineyards (see Additional File 2, Table S2). The resulting 99-sample dataset (11 vineyards, three developmental stages, three biological replicates, 1 year) showed a bimodal distribution of fluorescence intensity agreeing with the results of previous investigations [33]. To achieve a unimodal distribution from the whole dataset, we used k-means clustering of the log_2 _fluorescence intensities (see Additional File 9, Figure S4) applying increasing values of k until only a single cluster displayed bimodal distribution (k = 10) with a low mean expression level. We then grouped the nine unimodal clusters with high mean expression levels, allowing us to select genes providing a unimodal distribution without cutting off low-value expression data (for example, cluster 1, see Additional File 9, Figure S4). We identified 13,752 genes with a unimodal distribution of the fluorescence signal (see Additional File 10, Dataset S3). We carried out a Kruskal-Wallis test (*P *<0.01) on the reduced dataset from each vineyard to determine the number of genes that were differentially expressed during ripening and found the average number over the 11 vineyards was 8,381. Plastic genes modulated in at least one vineyard during ripening were identified by applying 11-group Kruskal-Wallis analysis to Dataset S3 (Additional File 10), resulting in a reduced set of 1,478 transcripts (*P *<0.01) (see Additional File 11, Dataset S4). The number of plastic genes appeared remarkably high (approximately 18% of the average number of modulated genes), suggesting that the ripening of Corvina berries can be modified extensively by the growing conditions. This also indicated that approximately 5% of the transcripts represented on the microarray correspond to plastic genes whose expression can vary under diverse growing conditions.

**Figure 3:**
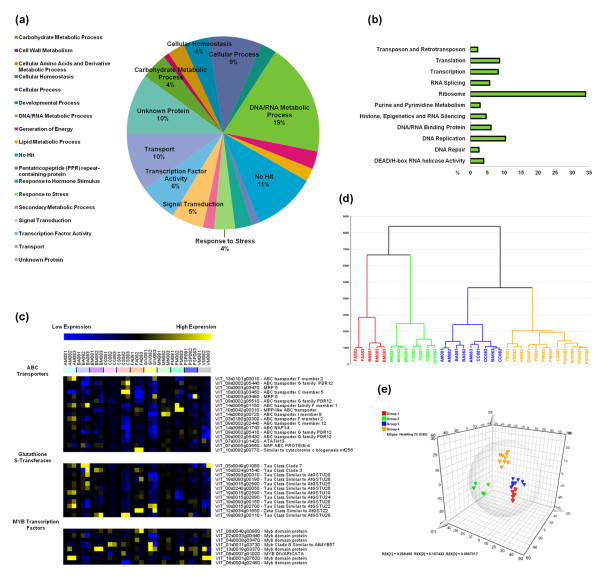
**Grapevine transcripts showing plasticity during berry development**. **(a) **Functional category distribution of the 1,478 (*P *<0.01) plastic grapevine genes. Transcripts were grouped into the 18 most represented functional categories, based on Plant GO Slim classification of biological processes. **(b) **Plant GO Slim classification of biological processes and functions for 280 transcripts in the 'DNA/RNA Metabolic Process' category. **(c) **Plastic members of the ABC transporter, glutathione-S-transferase, and MYB transcription factor gene families. The heat map of transcriptional profiles was generated with TMeV 4.8 using the average expression value of the three biological replicates. Sample names are composed by vineyard abbreviation followed by the indication of the harvesting year (08) and by the indication of the developmental stages (1, 2, or 3). **(d) **Principal component analysis using Simca P+ 12.0 (Umetrics). The PCA dendrogram was calculated using the average expression value of the three biological replicates. The dendrogram was designed using Ward's method and horizontally sorted by cluster size. Sample names are composed by vineyard abbreviation followed by the indication of the harvesting year (08) and by the indication of the developmental stages (1, 2, or 3). **(e) **Variables and scores three-dimensional scatter plot of the O2PLS-DA model (3+2+0, UV, R^2^X = 0.673, Q^2 ^= 0.775) applied to the 1,478 plastic transcripts dataset and colored according to the four-group partition as in the PCA analysis shown in (d). The model was created using Simca P+ (12.0). Components 3 and 2 represent the predictive and orthogonal components identified by the model, whereas 0 represents the background variation. UV: Unit variance scaling method.

The analysis of transcript functional categories revealed that 21% of the plastic genes were unrecognized ('No Hit') or uncharacterized ('Unknown Protein') suggesting that much remains to be learned about the genes expressed during berry development (Figure 3a). Overall, the 1,478 plastic transcripts were particularly enriched in the functional categories 'Translation', 'Nucleobase, nucleoside, nucleotide and nucleic acid metabolic process', 'Regulation of gene expression, epigenetic', and 'Transport' (see Additional File 12, Figure S5). In particular, at least 86 ribosomal proteins were found in the DNA/RNA metabolic process category (Figure 3b), suggesting that transcriptome reprogramming during ripening involves a shift in protein synthesis. 'Transcription factor activity' function is also well represented, for example, 30 zinc finger genes, including C(2)H(2)-type proteins that regulate stress and hormone response pathways [34] and many C3HC4-type RING zinc fingers that also play a role in abiotic stress responses [35,36]. We also identified at least eight members of the MYB transcription factor family (see the heat map in Figure 3c, which shows the expression profiles among vineyards and during ripening). Some members of the MYB family have been shown to regulate secondary metabolism in grape berries [37,38] as well as drought, salinity, and cold stress in Arabidopsis and rice [35,39].

Genes representing the 'Transport' functional category included those encoding ATP-binding cassette (ABC) proteins (Figure 3c). This is one of the largest and most diverse protein families in plants, and is responsible for transporting many different substances across membranes [40,41], suggesting a broad reprogramming of intracellular and intercellular transport as a component of phenotypic plasticity in Corvina berries. The glutathione S-transferase (GST) family was also well represented among the plastic genes, with at least 11 tau-class GSTs showing different expression patterns among the 11 vineyards (Figure 3c). Although the function of tau-class GSTs remains poorly understood, they may be involved in stress tolerance and secondary metabolism as well as the detoxification of herbicides [42]. It is noteworthy that many of the 'Response to stress' transcripts we identified are involved in ROS scavenging, such as two glutaredoxins, four ascorbate peroxidases, a nudix hydrolase, two peroxiredoxins, and three superoxide dismutases. Together with the many GSTs that reduce peroxides by controlling the balance between the oxidized and reduced forms of glutathione, the presence of these transcripts suggests that the oxidative burst observed in Pinot Noir berries at veraison [43] could also occur in Corvina and is part of the complex transcriptional rearrangement during berry plasticity. Finally, several of the Corvina plastic transcripts belonged to the 'Developmental process' category, including several homologs of Arabidopsis genes involved in floral transition and flower organ identity, that is, *EARLY FLOWERING*, *CONSTANS, FRIGIDA*, and *SEPALLATA *(see Additional File 11, Dataset S4).

We also investigated whether it is possible to identify groups of vineyards sharing specific pools of plastic transcripts. Principal component analysis (PCA) was applied to the 1,478 plastic genes and we identified five principal components explaining 67.4% of the variability. The resulting dendrogram highlighted four principal clusters of vineyards (Figure 3d). Samples from the same vineyard but from different developmental stages generally clustered in the same group, with the exception of five samples. FA081 and CS081 were outliers possibly because of the significant changes from veraison to later developmental stages. Samples from the GIV vineyard were also outliers, indicating a unique gene expression profile under these particular micro-environmental conditions. Plastic transcripts contributing to the definition of each statistical class were defined by applying a four-class orthogonal projections to latent structures discriminant analysis (O2PLS-DA) model to a 28-sample reduced dataset lacking the outlier samples (Figure 3e). The robustness of the model was tested by calculating the degree of overfitting (100 permutations) of the corresponding three-class PLS-DA model (see Additional File 13, Figure S6). We identified 53, 30, 33, and 29 transcripts specific for each cluster. Remarkably, the vineyards in cluster 1 were all characterized by the intensive transcription of genes encoding ribosomal proteins (almost half of the all the cluster-specific transcripts) (see Additional File 14, Dataset S5).

We next tested whether it was possible to associate specific transcripts to groups of vineyards sharing certain environmental attributes or using specific agricultural practices. We applied the Kruskal-Wallis approach (*P *<0.01) to the 13,752-unimodal-profiling-transcript dataset (see Additional File 10, Dataset S3) using in each case the appropriate number of groups (for example, two groups for the direction of rows, four groups for the rootstock type). Among all the combinations we tested, only the 'Trelling System' and the 'Geographical Area' categories gave statistically-validated results (see Additional File 15, Figure S7a and S7b). This indicated that the contribution of the four different rootstock genotypes have only a marginal impact on the plastic gene expression of berries compared to the other agricultural parameters and is not appreciable from our experimental design. We found that 373 transcripts (false discovery rate (FDR), 0.25%) were differentially-modulated between vineyards using a replacement cane Guyot system or a parral system. Interestingly, several transcripts encoding heat shock proteins and proteins that maintain membrane integrity were induced among vineyards using the Guyot system but not those using the parral system (see Additional File 15, Figure S7a, and see Additional File 16, Dataset S6). Transcripts associated with the macro-areas had more complex expression profiles. Of the 534 transcripts (FDR, 0.42%) found significant in the statistical test, only the absence of particular transcripts could be specifically assigned to the Soave, Bardolino, or Valpolicella areas (see Additional File 15, Figure S7b, and see Additional File 17, Dataset S7). Thus, the absence of these transcripts in one geographical area (and their presence in the other two) appears to be more important in the definition of transcriptomic plasticity among different cultivation areas.

### Transcriptomic grouping at harvest

We next focused on berry harvesting in 2008 because this was the most important from an agronomic perspective and allowed the relationship between transcriptome plasticity and cultivation micro-environment to be investigated in detail. We built a dataset from the fluorescence intensity values of 33 samples (11 vineyards, one developmental stage, three biological replicates, and 1 year) and carried out significance analysis of microarray (SAM) using a FDR of 0.1%. This revealed 11,323 significantly modulated transcripts. We focused on transcripts displaying a ≥2-fold change in at least one vineyard-to-vineyard comparison, narrowing the number of significant transcripts to 8,250 (see Additional File 18, Dataset S8). In order to determine inner dataset dynamics, a cluster dendrogram was built using Pearson's correlation values comparing the transcriptome from each sample, revealing two-cluster partitioning (see Additional File 19, Figure S8a). We then used t-test analysis (α = 0.05) to confirm the transcriptional separation between the two vineyards groups (see Additional File 19, Figure S8b). Functional category distribution analysis uncovered a profound difference in metabolism. Gene expression in the first group of vineyards (VM, GIV, CC, PM, AM, and FA) clearly depicted ripe berry samples (for example, a large number of transcripts related to secondary metabolism) whereas in the second group of vineyards (CS, PSP, BA, BM, and MN) photosynthesis-related genes were still actively transcribed (see Additional File 19, Figure S8c). This metabolic difference, confirmed also by classical berry maturation indexes (total acidity and °Brix/total acidity, see Additional File 19, Figure S8d) strongly indicates a disparity in the degree of ripening at harvesting.

**Figure 4:**
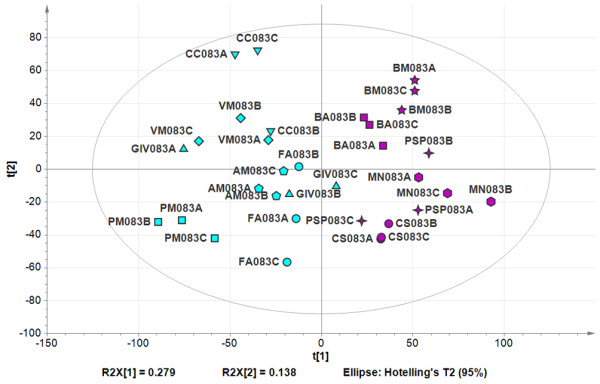
**Grapevine transcripts showing plasticity at harvest**. Principal component analysis of the whole third-stage dataset. The variables and scores scatter plot of the PCA model (nine components, R^2^X (cumulative) = 0.84, Q^2 ^(cumulative) = 0.602) was generated using Simca P+ 13.0 and colored according to the disparity in the degree of ripening, as illustrated in Figure S7C (Additional File 15). Different vineyards are indicated by different symbols. Sample names are composed by vineyard abbreviation, followed by the indication of the harvesting year (08), by the indication of the developmental stage (3), and by the description of the biological replicate (A, B, or C).

We applied PCA to the 8,250 differentially-modulated transcripts, and the first component, explaining 27.9% of the total dataset variability, was attributed to differences in ripening status as anticipated (Figure 4). This indicated that the plasticity of the berry transcriptome affected the entire berry ripening program, resulting in a diverse range of ripening characteristics at harvest. Overall, these data confirm that the phenotypic variation of grape berry responsible of the diverse qualitative traits a single clone can express in different growing sites reflects a deep plasticity of the berry transcriptome at harvest.

### Non-plastic berry genes

**Figure 5:**
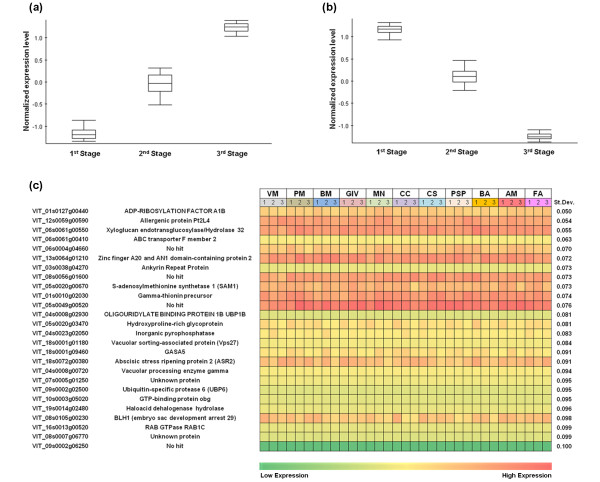
**Non-plastic grapevine genes**. Grape berry development markers. Box plots of the 115 most strongly upregulated **(a) **and the 90 most strongly downregulated genes **(b) **showing similar expression profiles in all vineyards. Box plots were created using Expander 6.0 [85]. The central line and outside edge of each box indicate the 50th, 25th, and 75th percentiles of expression data, respectively. Vertical lines on the two sides of the box represent the minimum and the maximum of all data, respectively. **(c) **Non-plastic constitutive genes. Genes with constant expression levels throughout berry development in all 11 different vineyards were ranked according to the lowest standard deviation among samples. The average expression value of the three biological replicates is indicated. The first 26 genes are shown (SD = 0.050-0.100).

The datasets also yielded developmental stage-specific but non-plastic transcripts, that is, those whose expression increases (positive markers) or declines (negative markers) with a constant profile during berry development regardless of the vineyard. This was achieved by applying SAM multiclass analysis (FDR, 0.1%, three groups) to the 99-sample dataset (11 vineyards, three developmental stages, three biological replicates, year 2008 only), revealing 18,190 transcripts that were differentially expressed among the three berry developmental stages but to the same extent in all 11 vineyards. These genes were likewise analyzed by one-way ANOVA (α = 0.01, three groups, standard Bonferroni correction) and the resulting 11,532 genes were grouped into eight k-means clusters of gene expression (Pearson's correlation). The clusters defined by a continuous increase or decline during ripening were further screened for genes with the largest fold change (95th percentile) between the first and last stages, to select those that were more strongly modulated. This yielded 115 upregulated genes (Figure 5a; see Additional File 20, Dataset S9) and 90 downregulated genes (Figure 5b; see Additional File 20, Dataset S9).

The non-plastic upregulated genes included those encoding pathogenesis related (PR) proteins and biotic stress factors such as thaumatins and osmotins, as previously reported [27,43-45]. The *PR10 *gene *VIT_05s0077g01530 *and two *PR1 *genes (*VIT_03s0088g00710 *and *VIT_03s0088g00690*) were previously shown to be differentially modulated during the last stages of berry development stages in Chardonnay grapes [46]. PR proteins are the most abundant proteins in wine and they are expressed across all stages of berry development [47]. The identification of PR-related transcripts as non-plastic developmental markers suggests that they represent a fundamental grapevine disease-prevention strategy that could help to avoid berry infections. We also identified eight non-plastic genes encoding germacrene-D-synthases and seven encoding stilbene synthases (see Additional File 20, Dataset S9) confirming previous reports that the terpene and phenylpropanoid pathways are under strict transcriptional control during ripening [48-50].

The non-plastic downregulated genes included many involved in photosynthesis, which occurs in the early berry until veraison [43,44,49]. We identified seven photosynthesis-related transcripts (mainly encoding polyphenol oxidases and photosystem II subunits) showing that the shutting down of photosynthesis can be used to monitor the progress of berry development regardless of the vineyard. Other non-plastic downregulated genes were involved in cell wall structural modifications, including transcripts for expansin A, xyloglucan endotransglucosylase/hydrolase (XTH), and β-D-xylosidase, agreeing with previous investigations of Chardonnay, Cabernet, and Corvina berries [43,44,46].

Finally, we identified a number of transcripts that were neither plastic (no variation among the 11 vineyards) nor developmentally modulated (no variation among the three developmental stages) using SAM multiclass analysis (FDR = 0.1%, 11 groups) of three stage-specific datasets, each comprising 33 samples (11 vineyards, one developmental stage, three biological replicates, 2008 season only). The constitutive and non-plastic transcripts were further analyzed by one-way ANOVA (α = 0.01, 11 groups). The 15,841, 14,342, and 13,286 transcripts that were constitutively expressed during veraison, mid-ripening, and ripening, respectively (see Additional File 21, Figure S9), were compared to identify 6,927 transcripts shared among all three developmental stages. These were screened for the lowest (last 99th percentile) standard deviation among samples, resulting in a set of 76 non-plastic genes constitutively expressed during berry development (Figure 5c; see Additional File 22, Dataset S10).

Transcripts scoring the lowest standard deviations included those encoding proteins related to intracellular transport (ADP-ribosylation factor, ABC transporter F member 2 and vacuolar sorting-associated protein), plant cell wall metabolism (xyloglucan endotransglucosylase/hydrolase), DNA and RNA binding and editing (zinc finger A20, AN1 domain-containing stress-associated protein 2 and oligouridylate-binding protein), and cellular metabolism (S-adenosylmethionine synthetase, inorganic pyrophosphatase, and ubiquitin-specific protease). Remarkably, five transcripts with different expression levels and different standard deviations showed constitutive expression also in the transcriptome of all grapevine organs (see Additional File 23, Figure S10), as confirmed in the recent grape gene expression atlas [33]. These 76 non-plastic constitutive genes are candidate reference genes for quantitative gene expression analysis.

## Discussion

The biological material at our disposal offered a unique opportunity to compare the same grapevine berry phenological phases in different vineyards and growth years, allowing us to correlate changes in the transcriptome with distinct growing conditions.

Our data suggest that veraison is a critical period during which the seasonal climate has its greatest effect whereas the microenvironment and agronomic practices had only a marginal impact (Figure 2a). The direct influence of climate on berry quality has been demonstrated, particularly the additive effects of temperature and water availability [51,52]. Many genes were differentially expressed among the years at veraison, with the greatest difference observed between the 2007 and 2008 seasons (Figure 2b). 2007 is characterized by the specific upregulation of genes related to disease resistance, abiotic stress adaptation and the oxidative burst, reflecting the severe stress imposed on this growth year by the high spring temperatures. The 2008 season is characterized by the significant upregulation of genes involved in DNA/RNA metabolic processes and transcription. The basis of this transcriptome reprogramming is hard to define since the climate was similar in the 2006 and 2008 seasons, but it may reflect a compensatory adaptation following the unusual 2007 season.

The strong effect of growth year on sample correlation faded during berry ripening (see Additional File 5, Figure S1a and S1b) suggesting that the impact of agronomic practices and environmental conditions on the berry transcriptome becomes more important at this stage. Nevertheless, we were still able to identify season-specific modulated genes at mid-ripening and at harvest. The major difference between the growth years involved secondary metabolism, particularly the broad expression of phenylpropanoid-related genes in 2006 and 2008 compared to 2007 berries. Indeed, we observed the induction of at least 13 phenylalanine ammonia lyases, (PALs), 43 stilbene synthases (STSs), 9 cinnamyl alcohol dehydrogenases (CADs), two cinnamoyl-CoA reductases (CCRs), and two caffeate 3-O-methyltransferases (COMTs) (Figure 2e; see Additional File 6, Dataset S2).

The synthesis of resveratrol and its derivatives in berries by STSs is stimulated by stress factors such as fungal infection (mainly *Botrytis cinerea*), wounding, and UV light [53,54]. However, it is becoming clear that higher levels of stilbenoid compounds and *STS *expression are also associated with the normal course of berry ripening in healthy and unstressed grapes [55-57]. Our data confirm that the increase in *STS *gene expression is likely to be a normal feature of grape ripening and distinguishes the ripening Corvina berries in typical climates from the unusual temperature in 2007 growing season. The same hold true for the expression behavior of *PAL *genes which are likely co-regulated with STSs during the biosynthesis of stilbenes, as previously reported [56,58].

The differential expression of genes (*CAD*, *CCR*, and *COMT*) involved in the metabolism of hydroxycinnamic acids, precursors of many volatile odor compounds, supports the idea that the aromatic profile of ripe berry is strongly influenced by the temperature condition during the growing season [51,59,60]. Our conclusion is supported by the lower amount of stilbenes like resveratrol and its derivatives (viniferins) and hydroxycinnamic compounds detected in 2007 berries compared to the other years (see Additional File 7, Figure S2).

The 2008 season showed the least plasticity of gene expression among different vineyards (see Additional File 8, Figure S3). We therefore used this year to broaden the analysis to 11 different vineyards, and we found that approximately 5% of transcripts on the microarray were modulated when Corvina berries were ripened under different environmental conditions and using different agronomical practices (see Additional File 11, Dataset S4). The limited number of available studies comprehensively describing transcriptome plasticity in plants makes it difficult to evaluate the percentage of plastic genes in the Corvina transcriptome accurately, but based on our datasets we found that plastic genes represented approximately 18% of genes modulated during ripening in the 11 vineyards, suggesting that the environment and agricultural practices can have a profound impact on the berry transcriptome, in turn affecting ripe berry and wine quality traits. Interestingly, approximately 27% of the plastic transcripts were 'commonly expressed' (that is, expressed in all organs and tissues in the plant) in the recent grapevine transcriptomic atlas [33] whereas approximately 73% were expressed in >30 plant organs/tissues and none were specifically expressed in berry tissues, suggesting that the plasticity of gene expression in grapevine is a broad phenomenon and that data representing the berry pericarp could also be used to study plasticity in other organs.

Many of the plastic genes (for example, ribosomal proteins and many other DNA/RNA metabolic process-related genes) we identified are worthy of further investigation to determine their specific impact on berry ripening parameters, for example, the modulation of ribosomal proteins suggests that transcriptome reprogramming during ripening involves a shift in protein synthesis (Figure 3a, b; see Additional File 11, Dataset S4 and Additional File 12, Figure S5). Although the regulation of ribosomal proteins in plants under different conditions has not been studied in detail, their modulation has been reported in response to various forms of abiotic stress including UV-B radiation [61], low temperatures [62,63], wounding [64], ozone radiation [65], and salinity [66]. Other genes in the DNA/RNA metabolic process category were related to stress responses and recovery, which often affects the transcription and translation of genes encoding ribosomal proteins and translation factors [67]. Our data strongly suggest that transcriptomic plasticity in developing Corvina berries is exerted predominantly by the broad reprogramming of genes that control the transcription and the rate of translation to remodel the cellular protein set.

Interestingly, we also identified several plastic transcripts putatively involved in floral transition and flower organ identity. These included transcripts encoding two EARLY FLOWERING homologs, a CONSTANS protein, and transcription factors such as FRIGIDA-LIKE 2, SUPPRESSOR OF FRIGIDA 4, and SEPALLATA 3 (see Additional File 11, Dataset S4). Although most of these genes are believed to be functionally conserved in grapevine [68-70], their precise roles remain to be determined because the grapevine latent bud develops continuously and is therefore distinct from both the herbaceous flowers of Arabidopsis and rice and the woody perennial model of poplar. Many floral development genes are photoperiod-dependent in grapevine and may also play a role in bud dormancy [71]. The MADS box transcriptional factor SEPALLATA 3, and the grapevine homologs of CONSTANS and EARLY FLOWERING 4, are positively regulated during berry development [68] and may help to determine berry weight [23]. Because fruits represent the continued growth of the ovary, we propose that these floral regulators play a critical role in berry development and plasticity.

Our analysis allowed us to define groups of vineyards sharing the expression profiles of common plastic genes (Figure 3d, e). Moreover, in some cases it was possible to link sets of differentially-expressed transcripts to particular environmental attributes or specific agronomical parameters (see Additional File 15, Figure S7a and S7b). Several heat-shock proteins have been found more expressed in Guyot-trained vines compared to the parral system. These genes have been detected as highly responsive to the microclimate changes around clusters [72]. Our data suggest that parrals are better shelters for berry clusters than replacement cane systems. Nevertheless, the inevitable absence of all possible combinations of environmental and agricultural parameters for plants cultivated in the open field means that our investigation could only provide an exploratory perspective rather than predictive interpretation.

Differential gene expression in fully-ripe Corvina berries highlighted a deep metabolic difference among samples harvested in different locations (Figure 4; see Additional File 19, Figure S8). We found a positive correlation between transcriptomic data and ripening parameters (see Additional File 19, Figure S8d) confirming that plasticity affects the entire maturation process, therefore candidate genes representing such plasticity (that is, photosynthesis-related and secondary metabolism-related genes) could eventually be used for on-field monitoring.

The large scale of our sampling procedure also allowed the identification of genes that were not plastic, that is, genes that were either constitutive or developmentally regulated but whose expression profiles were constant over the different vineyards and cultivation environments. Developmentally regulated but non-plastic genes (see Additional File 20, Dataset S9) included several positive and negative markers that have previously been identified as differentially-modulated transcripts during berry development in other seasons (2003 to 2006) and in other varieties (Chardonnay, Cabernet Sauvignon, and Pinot Noir) [43,44,46]. These could be developed into universal markers suitable for the monitoring of grape ripening in the field, regardless of cultivar and environment. The constitutive non-plastic genes we identified (see Additional File 22, Dataset S10) add to the list of constitutive housekeeping that can be used as references during quantitative gene expression analysis, and have been validated by comparison with the recent grapevine atlas of gene expression [33].

## Conclusions

Climate change is expected to significantly impact agriculture in the near future and poses serious threats, especially to those specialty crops, as grapevine, that are more valued for their secondary metabolites rather than for high yield. Phenotypic plasticity is believed to effectively buffer environmental extremes and maintain homeostasis of primary metabolism.

Overall, we have used the grapevine genome sequence [24] and the NimbleGen microarray platform to map the Corvina berry transcriptome and determine which genes are plastic (modulated in response to different environments) and which are non-plastic (regulated in the same manner regardless of the environment). This is the first major and comprehensive study to chart the plastic transcriptome in a woody perennial plant and our data therefore provide a reference model to explore genotype per environment interactions in fruit crops.

These new findings, together with the earlier transcriptomic, proteomic, and metabolomic studies focusing on the Corvina cultivar [25,33,45,49,50,57,73], provide a valuable platform to study the molecular processes underlying the complex development of grape berries and to identify environmentally-dependent and agriculturally-important traits which are essential for breeding new cultivars with improved adaptation to the environment. The methods used to establish our model provide a framework for the analysis of transcriptome plasticity in other crops as they respond to diverse environments and agricultural practices.

## Materials and methods

### Plant material

*Vitis vinifera *cv Corvina clone 48 berries were harvested from 11 different vineyards near Verona, Italy. We harvested 30 clusters from different positions along two vine rows and from random heights and locations on the plant to ensure the entire vineyard was represented. Samples of berries were harvested at three developmental stages (veraison, mid-ripening, and harvesting time) within 1 day in all 11 vineyards we investigated. Three berries were randomly selected from each cluster, avoiding those with visible damage and/or signs of pathogen infection. The berries were frozen immediately in liquid nitrogen. The °Brix of the must was determined using a digital DBR35 refractometer (Giorgio Bormac, Italy).

### Meteorological data

Meteorological data were kindly provided by the Veneto Regional Agency for Prevention and Protection (ARPAV). Temperature measurements were obtained from three recording stations in the macro-areas studied in this project (Illasi - Soave, Marano di Valpolicella - Valpolicella, Villafranca di Verona - Bardolino). Average daily temperature measurements were used to define average monthly temperatures and seasonal temperature trends. No significant differences were found among the three locations and averaged values were therefore used for Figure 1b.

### RNA extraction

Total RNA was extracted from approximately 400 mg of berry pericarp tissue (entire berries without seeds) ground in liquid nitrogen, using the Spectrum™ Plant Total RNA kit (Sigma-Aldrich, St. Louis, MO, USA) with some modifications [33]. RNA quality and quantity were determined using a Nanodrop 2000 spectrophotometer (Thermo Scientific, Wilmington, DE, USA) and a Bioanalyzer Chip RNA 7500 series II (Agilent, Santa Clara, CA, USA).

### Microarray analysis

We hybridized 10 μg of total RNA per sample to a NimbleGen microarray 090818_Vitus_exp_HX12 chip (Roche, NimbleGen Inc., Madison, WI, USA), which contains probes representing 29,549 predicted grapevine genes [74] covering approximately 98.6% of the genes predicted in the V1 annotation of the 12X grapevine genome [75]. Each microarray was scanned using an Axon GenePix 4400A (Molecular Devices, Sunnyvale, CA, USA) at 532 nm (Cy3 absorption peak) and GenePix Pro7 software (Molecular Devices) according to the manufacturer's instructions. Images were analyzed using NimbleScan v2.5 software (Roche), which produces Pair Files containing the raw signal intensity data for each probe and Calls Files with normalized expression data derived from the average of the intensities of the four probes for each gene. In the case of gene families and paralog genes, the specificity of the probe set for each single gene was assessed to exclude the possibility of cross-hybridization signals [33]. All microarray expression data are available at GEO under the series entry GSE41633 [76].

### Statistical analysis

Correlation matrixes were prepared using R software and Pearson's correlation coefficient as the statistical metric to compare the values of the whole transcriptome in all analyzed samples using the average value of the three biological replicates (29,549 genes). Correlation values were converted into distance coefficients to define the height scale of the dendrogram.

Hierarchical cluster analysis (HCL) and k-means cluster (KMC) analysis was applied using Pearson's correlation distance (TMeV 4.8 [77]).

The choice between parametric (t-test and ANOVA) and non-parametric (Kruskall-Wallis) analysis was made according to the unimodal or bimodal distribution of fluorescence intensities in each particular dataset (TMeV 4.8 [77]).

### Functional category distribution and GO enrichment analysis

All transcripts were annotated against the V1 version of the 12X draft annotation of the grapevine genome [78] allowing 70% of the genes to be identified. This was verified manually and integrated using Gene Ontology (GO) classifications. Transcripts were then grouped into the 15 highly-represented functional categories (GO:0009987, Cellular Processes; GO:0051090, Transcription Factor Activity; GO:0009725, Response to Hormone Stimulus; GO:0019725, Cellular Homeostasis; GO:0007165, Signal Transduction; GO:0006950, Response to Stress; GO:0032502, Developmental Process; GO:0006810, Transport; GO:0006091, Generation of Energy; GO:0090304, DNA/RNA Metabolic Process; GO:0044036, Cell Wall Metabolism; GO:0019748, Secondary Metabolic Process; GO:0006629, Lipid Metabolic Process; GO:0006520, Cellular Amino Acids and Derivative Metabolic Process; GO:0005975, Carbohydrate Metabolic Process), based on GO biological processes. Genes encoding pentatricopeptide (PPR) repeat-containing proteins and genes with unknown functions or with 'No Hit' annotations were also included.

GO enrichment analysis was applied to the 1,478 plastic genes using the BiNGO 2.3 plug-in tool in Cytoscape version 2.6 with PlantGOslim categories, as described by Maere *et al. *[79]. Over-represented PlantGOslim categories were identified using a hypergeometric test with a significance threshold of 0.1.

### Visualization of grapevine transcriptomics data using MapMan software

Information from the Nimblegen microarray platform was integrated using MapMan software [32] as described for the Array Ready Oligo Set *Vitis vinifera *(grape), the AROS V1.0 Oligo Set (Operon, Qiagen), and the GeneChip^® ^*Vitis vinifera *Genome Array (Affymetrix) [80].

### Principal component analysis (PCA) and orthogonal partial least squares (O2PLS) discriminant analysis

Principal component analysis (PCA) was carried out using SIMCA P+ 12 software (Umetrics, USA). O2PLS-DA was used to find relationships between two transcriptome datasets (X and Y) by decomposing the systematic variation in the X-block or Y-block into two model parts (a predictive part, which models the joint X-Y correlated variation, and an orthogonal part, which is not related to Y or X). The latent structures of the joint X-Y correlated variation were used to identify small groups of correlated variables belonging to the two different blocks by evaluating the similarity between each variable and the predictive latent components of the X-Y O2PLS model by means of their correlation. In order to set the significance threshold for the similarity, a permutation test was carried out, and data integration was performed on each small group of X-Y variables with significant correlation. O2PLS-DA allowed the identification of latent variables that were able to yield a parsimonious and efficient representation of the process. In order to define the number of latent components for OPLS-DA models, we applied partial cross-validation and a permutation test to reveal overfitting. Multivariate data analysis was performed by using SIMCA P+ 12 (Umetrics, USA).

### Metabolomics analysis

The same powdered samples used for RNA extraction were extracted in three volumes (w/v) of methanol acidified with 0.1 % of formic acid (v/v) in an ultrasonic bath at room temperature and 40 kHz for 15 min.

HPLC-ESI-MS was carried out using a Beckman Coulter Gold 127 HPLC system (Beckman Coulter, Fullerton, CA, USA) equipped with a System Gold 508 Beckman Coulter autosampler (Beckman Coulter, Fullerton, CA, USA). Metabolites were separated on an analytical Alltima HP RP-C18 column (150 × 2.1 mm, particle size 3 μm) equipped with a C18 guard column (7.5 × 2.1 mm) both purchased from Alltech (Alltech Associates Inc, Derfield, IL, USA). Two solvents were used: solvent A (5% (v/v) acetonitrile, 5% (v/v) formic acid in water), and solvent B (100% acetonitrile). The linear gradient, at a constant flow rate of 0.2 mL/min, was established from 0 to 10% B in 5 min, from 10 to 20% B in 20 min, from 20 to 25% B in 5 min, and from 25 to 70% B in 15 min. Each sample was analyzed in duplicate, with a 30 μL injection volume and 20-min re-equilibration between each analysis.

Mass spectra were acquired using a Bruker ion mass spectrometer Esquire 6000 (Bruker Daltonik GmbH, Bremen, Germany) equipped with an electrospray ionization source. Alternate negative and positive ion spectra were recorded in the range 50 m/z to 1,500 m/z (full scan mode, 13,000 m/z s^-1^). For metabolite identification, MS/MS and MS^3 ^spectra were recorded in negative or positive mode in the range 50 m/z to 1500 m/z with a fragmentation amplitude of 1 V. Nitrogen was used as the nebulizing gas (50 psi, 350°C) and drying gas (10 L/min). Helium was used as the collision gas. The vacuum pressure was 1.4 × 10-5 mbar. Additional parameters were: capillary source, +4,000 V; end plate offset, -500 V; skimmer, -40 V; cap exit, -121 V; Oct 1 DC, -12 V; Oct 2 DC, -1.70 V; lens 1, 5 V; lens 2, 60 V; ICC for positive ionization mode, 20,000; ICC for negative ionization mode; 7,000.

MS data were collected using the Bruker Daltonics Esquire 5.2-Esquire Control 5.2 software, and processed using the Bruker Daltonics Esquire 5.2-Data Analysis 3.2 software (Bruker Daltonik GmbH, Bremen, Germany). Metabolites were identified by comparison of m/z values, fragmentation patterns (MS/MS and MS^3^), and retention times of each signal with those of available commercial standards and by comparison of data previously published by our group [25,49]. Matrix effect did not affect relative quantification under these analysis conditions (data not shown) as previously demonstrated [25].

All metabolomics data are available in the Metabolights database under the series entry MTBLS39 [81].

### Enological analyses

Three replicates of 20 berry samples were crushed and the resulting must was clarified by centrifugation. Total acidity (expressed in g/L of tartaric acid) was quantified according to the *Compendium of international methods of Wine and Must analysis *- Office International de la Vigne et du vin [82]. Another three replicates of the 20 berry samples were crushed and analyzed according to the Glories method [83] to determine total anthocyanin levels.

## Abbreviations

FDR: False discovery rate; GO, Gene Ontology; LC-ESI-MS: Liquid chromatography - electrospray ionization-mass spectrometry; O2PLS-DA: Orthogonal projections to latent structures discriminant analysis; PCA: Principal component analysis; SAM: Significance analysis of microarray

## Competing interests

The authors declare that they have no competing interests.

## Authors' contributions

SDS performed the microarray studies and the data analysis, interpreted bioinformatic results, and drafted the manuscript. GBT and SZ participated in the design of the study and in the data analysis, and drafted the manuscript. MF participated in interpreting bioinformatic results and drafted the manuscript. LF participated in the data analysis. AA and FG performed LC-ESI-MS studies and interpreted metabolomics results. MD provided microarray platform technical support. MP supervised and coordinated the study. All authors carefully read and approved the final manuscript.

## Supplementary Material

Additional File 1 **Table S1**. Description of Corvina clone 48 grape collection sites, listing geographical parameters, farming, and agricultural practices. a.s.l: above sea level. Click here for file

Additional File 2 **Table S2**. Description of sample names sorted by year of harvesting. Names are composed by vineyard abbreviations, followed by the indication of the harvesting year (06, 07, or 08), by the indication of the developmental stage (1, 2, or 3) and by the description of the biological replicate (A, B, or C). When the biological replicate is not indicated, names are referred to the average of the three replicates. Click here for file

Additional File 3 **Table S3**. Maturation parameters of samples used for microarray analysis sorted by year of harvesting. Values represent mean ± standard deviation of three biological replicates. Total acidity is expressed in g/L of tartaric acid. For metabolic parameters, values are expressed as mean peak area ± standard deviation of three biological replicates. Click here for file

Additional File 4 **Dataset S1**. Four clusters of genes differentially modulated among the 2006, 2007, and 2008 seasons at veraison. Expression was measured as the average log_2 _intensity of each biological triplicate. Each value has been normalized on the median value of each row/gene. Click here for file

Additional File 5 **Figure S1**. Cluster dendrogram of **(a) **the second developmental stage and **(b) **the third developmental stage datasets using the average expression value of the three biological replicates. The Pearson's correlation values were converted into distance coefficients to define the height of the dendrograms. Blue, green, and red indicate samples harvested in 2006, in 2008, and in 2007, respectively. Click here for file

Additional File 6 **Dataset S2**. Genes that are differentially expressed between average climate seasons (2006 to 2008) and an exceptionally warm spring (2007) in second and third ripening time point samples. Transcripts modulated also at veraison (Dataset S1) are highlighted in the column 'Cluster Figure 2B'. Expression was measured as the average log_2 _intensity of each biological triplicate. Each value has been normalized on the median value of each row/gene. Click here for file

Additional File 7 **Figure S2**. Differential accumulation of metabolites between the 2006/2008 and 2007 vintages. Values were calculated as mean peak area ± standard deviation of three biological replicates and are expressed as fold-change of vintages 2006 to 2008 compared to 2007. Click here for file

Additional File 8 **Figure S3**. Plastic and vintage-specific transcripts. Kruskal-Wallis non-parametric variance analysis was carried out three times (*P *<0.05, four groups) on each vintage-specific dataset to obtain differentially-modulated genes among the four vineyards studied in each year. The Venn diagram was constructed using Venn [84] and redrawn. Click here for file

Additional File 9 **Figure S4**. k-means clustering of fluorescence log_2 _intensities. Increasing values of k were used until only one cluster displayed bimodal distribution (k = 10) with a low expression level mean value. Click here for file

Additional File 10 **Dataset S3**. Set of 13,752 transcripts with a unimodal expression profile in the 2008-harvested samples. Expression was measured as the average log_2 _intensity in all developmental stages and in all biological triplicates. Click here for file

Additional File 11 **Dataset S4**. Functional categories and expression values of 1478 plastic transcripts in 2008. The Kruskal-Wallis Statistic (H) and *P *value are indicated for each transcript. Expression was measured as the average intensity of each biological triplicate. Click here for file

Additional File 12 **Figure S5**. Enriched GO terms for the 1,478 plastic genes listed in Dataset S4. The network graphs show BiNGO visualizations of the overrepresented GO terms. Categories in GoSlimPlants [79] were used to simplify this analysis. Non-colored nodes are not over-represented, but they may be the parents of overrepresented terms. Node size is positively correlated with the number of genes belonging to the category. Colored nodes represent GO terms that are significantly over-represented (*P *value <0.1), with the shade indicating significance as shown in the color bar. Click here for file

Additional File 13 **Figure S6**. Validation of O2PLS-DA model. The three-latent-component O2PLS-DA model in Figure 3E was partially cross-validated and a permutation test (100 permutations) was used to highlight putative overfitting. Click here for file

Additional File 14 **Dataset S5**. Functional categories of specific transcripts from each of four O2PLS-DA clusters. Pq(corr) values are indicated for each direction. Click here for file

Additional File 15 **Figure S7**. Hierarchical clustering analysis of environment-specific differentially expressed genes in **(a) **vineyards using parral or Guyot replacement cane trelling systems and **(b) **vineyards located in one of the three macro-areas we investigated. Pearson's correlation distance was used as the metric. The heat map of transcriptional profiles was generated with TMeV 4.8 using the average expression value of the three biological replicates per each developmental stage. Click here for file

Additional File 16 **Dataset S6**. Differentially-expressed genes in the 'Trelling System' category. Expression was measured as the average log_2 _intensity of each biological triplicate. Each value has been normalized on the median value of each row/gene. Click here for file

Additional File 17 **Dataset S7**. Differentially-expressed genes in the 'Geographical Area' category. Expression was measured as the average log_2 _intensity of each biological triplicate. Each value has been normalized on the median value of each row/gene. Click here for file

Additional File 18 **Dataset S8**. Plastic transcripts from the grape berry transcriptome at harvesting in 2008. Click here for file

Additional File 19 **Figure S8**. Differentially modulated genes at harvesting. **(a) **Cluster dendrogram of the third developmental stage dataset using the average expression value of the three biological replicates. Pearson's correlation values were converted into distance coefficients to define the height of the dendrogram. Different colors indicate the disparity in the degree of ripening as analyzed in **(c)**. **(b) **Differentially-expressed genes between the two groups of vineyards highlighted in (a). A t-test (α = 0.05) was performed between the two groups of vineyards, and a k-means analysis was computed using Pearson's distance to generate the line plots. **(c) **Functional category distribution of the differentially-modulated genes between the two groups of vineyards during harvesting. Transcripts were grouped into the 18 most represented functional categories, based on Plant GO Slim classification of biological processes. Sample VM083, GIV083, CC083, PM083, AM083, and FA083 category distribution is depicted in purple, while sample CS083, PSP083, BA083, BM083, and MN083 category distribution is depicted in light green. **(d) **Total acidity, expressed in g/L of tartaric acid and °Brix/total acidity of samples from the third developmental stage. Values represent mean ± standard deviation of three biological replicates. Different colors indicate the disparity in the degree of ripening as shown in (a) and in (c). Click here for file

Additional File 20 **Dataset S9**. Markers for grape berry development. Markers also showing significance in other vintages are labeled accordingly. Click here for file

Additional File 21 **Figure S9**. Non-plastic genes. Stage-specific datasets were analyzed by SAM multiclass analysis and one-way ANOVA (11 groups). Transcripts not shown to be significant in either analysis (that is, not differentially modulated) were tested for stage-specificity. The Venn diagram was calculated using Venn [84] and redrawn. Click here for file

Additional File 22 **Dataset S10**. Non-plastic and constitutive transcripts. Expression was measured as the average log_2 _intensity of each biological triplicate. Click here for file

Additional File 23 **Figure S10**. Expression profile of five non-plastic constitutive genes in the whole grapevine expression atlas [33]. Click here for file
