# Supplementary material for: The plasticity of the grapevine berry transcriptome
Source: Genome Biol. 2013 Jun 7;14(6):r54. doi: 10.1186/gb-2013-14-6-r54 (PMC3706941; doi:10.1186/gb-2013-14-6-r54)
Supplement: Additional File 1 — Table S1. Description of Corvina clone 48 grape collection sites, listing geographical parameters, farming, and agricultural practices. a.s.l: above sea level. [file gb-2013-14-6-r54-S1.PDF]

Table S1 Description of Corvina clone 48 grape collection sites.

Vineyard	Location	Area	Vineyard Site	Altitude (m a.s.l.)[1]	Type of Soil (% Sand, % Clay, % Silt)	Vineyard Training System	Rows Facing Direction	Planting Layout (m)[2]	Vineyard Age (y)[3]	Rootstock
**AM**	Marcellise	Soave	45°27′ 23″ N ; 11°05′ 52″ E	250	15, 43, 42	Parral	East - West	3.20x100	8	41B
**BA**	Affi	Bardolino	45°33′ 23″ N ; 10°47′ 50″ E	120	47, 36, 17	Parral	North - South	4.50x0.80	13	SO4
**BM**	Marano	Valpollicella	45°33′ 33″ N ; 10°54′ 15″ E	450	66, 21, 13	Guyot	East - West	4.00x125	18	K55B
**CC**	Colognola	Soave	45°26′ 30″ N ; 11°11′ 42″ E	120	35, 29, 36	Guyot	North - South	2.50x0.90	6	K55B
**CS**	Sona	Bardolino	45°25′ 51″ N ; 10°49′ 11″ E	100	42, 37, 21	Parral	East - West	3.50x120	10	420A
**FA**	Arbizzano	Valpollicella	45°29′ 41″ N ; 10°56′ 42″ E	130	29, 39, 32	Parral	East - West	3.50x0.75	9	420A
**GIV**	Cavaion	Bardolino	45°31′ 23″ N ; 10°46′ 06″ E	120	53, 32, 15	Parral	East - West	3.00x100	15	SO4
**MN**	Negrar	Valpollicella	45°33′ 11″ N ; 10°57′ 16″ E	250	13, 67, 20	Guyot	North - South	2.80x120	8	K55B
**PM**	Montorio	Soave	45°27′ 17″ N ; 11°03′ 14″ E	130	36, 36, 28	Guyot	North - South	180x0.80	7	41B
**PSP**	S.Pietro Incariano	Valpollicella	45°30′ 24″ N ; 10°53′ 12″ E	100	40, 32, 28	Guyot	North - South	2.80x100	6	K55B
**VM**	Sommacampagna	Bardolino	45°25′ 10″ N ; 10°50′ 20″ E	100	49, 33, 18	Guyot	East - West	2.30x0.90	7	K55B

[1] Meters above sea level

[2] Meters

[3] Years

Table S1

Table S1. Description of Corvina clone 48 grape collection sites, listing geographical parameters, farming and agricultural practices (a.s.l. = above sea level).
