# Supplementary material for: The plasticity of the grapevine berry transcriptome
Source: Genome Biol. 2013 Jun 7;14(6):r54. doi: 10.1186/gb-2013-14-6-r54 (PMC3706941; doi:10.1186/gb-2013-14-6-r54)
Supplement: Additional File 2 — Table S2. Description of sample names sorted by year of harvesting. Names are composed by vineyard abbreviations, followed by the indication of the harvesting year (06, 07, or 08), by the indication of the developmental stage (1, 2, or 3) and by the description of the biological replicate (A, B, or C). When the biological replicate is not indicated, names are referred to the average of the three replicates. [file gb-2013-14-6-r54-S2.PDF]

2006

Developmental Stage	1			2			3		
Biological Replicate	A	B	C	A	B	C	A	B	C
AM	AM 06 1 A	AM 06 1 B	AM 06 1 C	AM 06 2 A	AM 06 2 B	AM 06 2 C	AM 06 3 A	AM 06 3 B	AM 06 3 C
CS	CS 06 1 A	CS 06 1 B	CS 06 1 C	CS 06 2 A	CS 06 2 B	CS 06 2 C	CS 06 3 A	CS 06 3 B	CS 06 3 C
MN	MN 06 1 A	MN 06 1 B	MN 06 1 C	MN 06 2 A	MN 06 2 B	MN 06 2 C	MN 06 3 A	MN 06 3 B	MN 06 3 C
PSP	PSP 06 1 A	PSP 06 1 B	PSP 06 1 C	PSP 06 2 A	PSP 06 2 B	PSP 06 2 C	PSP 06 3 A	PSP 06 3 B	PSP 06 3 C

2007

Developmental Stage	1			2			3		
Biological Replicate	A	B	C	A	B	C	A	B	C
AM	AM 07 1 A	AM 07 1 B	AM 07 1 C	AM 07 2 A	AM 07 2 B	AM 07 2 C	AM 07 3 A	AM 07 3 B	AM 07 3 C
CS	CS 07 1 A	CS 07 1 B	CS 07 1 C	CS 07 2 A	CS 07 2 B	CS 07 2 C	CS 07 3 A	CS 07 3 B	CS 07 3 C
MN	MN 07 1 A	MN 07 1 B	MN 07 1 C	MN 07 2 A	MN 07 2 B	MN 07 2 C	MN 07 3 A	MN 07 3 B	MN 07 3 C
PSP	PSP 07 1 A	PSP 07 1 B	PSP 07 1 C	PSP 07 2 A	PSP 07 2 B	PSP 07 2 C	PSP 07 3 A	PSP 07 3 B	PSP 07 3 C

2008

Developmental Stage	1			2			3		
Biological Replicate	A	B	C	A	B	C	A	B	C
AM	AM 08 1 A	AM 08 1 B	AM 08 1 C	AM 08 2 A	AM 08 2 B	AM 08 2 C	AM 08 3 A	AM 08 3 B	AM 08 3 C
BA	BA 08 1 A	BA 08 1 B	BA 08 1 C	BA 08 2 A	BA 08 2 B	BA 08 2 C	BA 08 3 A	BA 08 3 B	BA 08 3 C
BM	BM 08 1 A	BM 08 1 B	BM 08 1 C	BM 08 2 A	BM 08 2 B	BM 08 2 C	BM 08 3 A	BM 08 3 B	BM 08 3 C
CC	CC 08 1 A	CC 08 1 B	CC 08 1 C	CC 08 2 A	CC 08 2 B	CC 08 2 C	CC 08 3 A	CC 08 3 B	CC 08 3 C
CS	CS 08 1 A	CS 08 1 B	CS 08 1 C	CS 08 2 A	CS 08 2 B	CS 08 2 C	CS 08 3 A	CS 08 3 B	CS 08 3 C
FA	FA 08 1 A	FA 08 1 B	FA 08 1 C	FA 08 2 A	FA 08 2 B	FA 08 2 C	FA 08 3 A	FA 08 3 B	FA 08 3 C
GIV	GIV 08 1 A	GIV 08 1 B	GIV 08 1 C	GIV 08 2 A	GIV 08 2 B	GIV 08 2 C	GIV 08 3 A	GIV 08 3 B	GIV 08 3 C
MN	MN 08 1 A	MN 08 1 B	MN 08 1 C	MN 08 2 A	MN 08 2 B	MN 08 2 C	MN 08 3 A	MN 08 3 B	MN 08 3 C
PM	PM 08 1 A	PM 08 1 B	PM 08 1 C	PM 08 2 A	PM 08 2 B	PM 08 2 C	PM 08 3 A	PM 08 3 B	PM 08 3 C
PSP	PSP 08 1 A	PSP 08 1 B	PSP 08 1 C	PSP 08 2 A	PSP 08 2 B	PSP 08 2 C	PSP 08 3 A	PSP 08 3 B	PSP 08 3 C
VM	VM 08 1 A	VM 08 1 B	VM 08 1 C	VM 08 2 A	VM 08 2 B	VM 08 2 C	VM 08 3 A	VM 08 3 B	VM 08 3 C

Table S2

Table S2. Description of sample names sorted by year of harvesting. Names are composed by vineyard abbreviations, followed by the indication of the harvesting year (06, 07 or 08), by the indication of the developmental stage (1, 2 or 3) and by the description of the biological replicate (A, B or C). When the biological replicate is not indicated, names are referred to the average of the three replicates.
