# Supplementary material for: The plasticity of the grapevine berry transcriptome
Source: Genome Biol. 2013 Jun 7;14(6):r54. doi: 10.1186/gb-2013-14-6-r54 (PMC3706941; doi:10.1186/gb-2013-14-6-r54)
Supplement: Additional File 3 — Table S3. Maturation parameters of samples used for microarray analysis sorted by year of harvesting. Values represent mean ± standard deviation of three biological replicates. Total acidity is expressed in g/L of tartaric acid. For metabolic parameters, values are expressed as mean peak area ± standard deviation of three biological replicates. [file gb-2013-14-6-r54-S3.PDF]

Table S3 Maturation parameters of samples used for microarray analyses sorted by harvesting year.

	° BRIX			Total Anthocyanins			Total Acidity			Qglc / Qglr		
	Developmental Stage			Developmental Stage			Developmental Stage			Developmental Stage		
	1	2	3	1	2	3	1	2	3	1	2	3
**AM 06**	n.m.	18.6 ± 0.6	21.5 ± 1.0	61.38 ± 36.11	377.93 ± 21.08	384.11 ± 16.63	22.39 ± 0.05	10.19 ± 0.04	6.52 ± 0.01	0.21 ± 0.02	0.75 ± 0.16	1.28 ± 0.15
**CS 06**	n.m.	18.6 ± 0.7	21.4 ± 0.8	40.38 ± 14.11	473.02 ± 150.72	504.68 ± 125.94	30.78 ± 0.04	12.28 ± 0.01	8.90 ± 0.06	0.11 ± 0.02	0.70 ± 0.12	1.61 ± 0.33
**MN 06**	n.m.	17.5 ± 1.1	17.1 ± 0.5	5.07 ± 0.48	625.37 ± 90.93	455.66 ± 91.91	37.10 ± 0.03	11.90 ± 0.00	7.76 ± 0.06	0.11 ± 0.02	1.11 ± 0.36	1.67 ± 0.13
**PSP 06**	n.m.	17.8 ± 0.3	20.4 ± 0.6	20.15 ± 1.09	268.74 ± 66.78	323.77 ± 139.39	28.56 ± 0.06	9.86 ± 0.01	6.83 ± 0.01	0.22 ± 0.07	1.11 ± 0.38	1.32 ± 0.43

n.m. = not measured

	° BRIX			Total Anthocyanins			Total Acidity			Qglc / Qglr		
	Developmental Stage			Developmental Stage			Developmental Stage			Developmental Stage		
	1	2	3	1	2	3	1	2	3	1	2	3
**AM 07**	n.m.	13.4 ± 0.4	18.8 ± 0.7	5.05 ± 2.16	238.84 ± 97.16	363.79 ± 162.24	30.56 ± 0.28	8.38 ± 0.06	6.43 ± 0.00	0.16 ± 0.12	1.06 ± 0.31	1.63 ± 0.22
**CS 07**	n.m.	14.2 ± 0.4	18.2 ± 1.0	13.95 ± 16.43	490.65 ± 63.54	325.26 ± 44.59	32.97 ± 0.22	9.66 ± 0.25	6.91 ± 0.01	0.16 ± 0.08	0.80 ± 0.07	1.59 ± 0.17
**MN 07**	n.m.	10.0 ± 0.6	16.6 ± 2.5	5.07 ± 2.30	99.49 ± 29.38	566.72 ± 24.96	40.51 ± 0.30	16.10 ± 0.02	8.50 ± 0.06	0.11 ± 0.02	0.43 ± 0.11	1.33 ± 0.07
**PSP 07**	n.m.	13.59 ± 0.3	19.52 ± 1.7	n.m.	n.m.	n.m.	38.35 ± 0.02	9.25 ± 0.04	5.68 ± 0.02	n.m.	n.m.	n.m.

n.m. = not measured

	° BRIX			Total Anthocyanins			Total Acidity			Qglc / Qglr		
	Developmental Stage			Developmental Stage			Developmental Stage			Developmental Stage		
	1	2	3	1	2	3	1	2	3	1	2	3
**AM 08**	n.m.	16.0 ± 0.5	19.9 ± 0.3	155.43 ± 14.89	420.05 ± 44.91	586.11 ± 34.85	39.66 ± 0.67	19.20 ± 0.04	9.55 ± 0.10	0.15 ± 0.07	1.08 ± 0.15	1.43 ± 0.07
**BA 08**	n.m.	15.1 ± 1.1	17.8 ± 1.0	52.49 ± 23.51	461.70 ± 64.49	615.42 ± 143.20	34.00 ± 0.32	31.66 ± 0.04	13.14 ± 0.01	0.10 ± 0.02	1.02 ± 0.20	1.59 ± 0.49
**BM 08**	n.m.	13.8 ± 1.0	20.2 ± 1.6	17.18 ± 9.33	369.90 ± 39.80	546.15 ± 21.43	33.40 ± 0.03	37.80 ± 0.00	16.61 ± 0.07	0.10 ± 0.02	0.80 ± 0.23	1.65 ± 0.16
**CC 08**	n.m.	16.6 ± 0.8	20.3 ± 1.8	185.08 ± 33.10	437.35 ± 68.84	442.40 ± 95.96	37.24 ± 0.16	20.61 ± 0.04	7.92 ± 0.02	0.66 ± 0.08	1.95 ± 0.45	2.44 ± 0.73
**CS 08**	n.m.	16.4 ± 1.3	19.7 ± 2.0	182.56 ± 78.49	549.60 ± 20.23	543.58 ± 18.42	37.04 ± 0.16	17.51 ± 0.11	7.54 ± 0.00	0.20 ± 0.04	1.08 ± 0.53	1.53 ± 0.06
**FA 08**	n.m.	15.8 ± 0.6	17.7 ± 0.3	169.44 ± 40.04	340.53 ± 62.86	355.25 ± 61.13	40.82 ± 0.52	11.81 ± 0.15	6.28 ± 0.00	0.51 ± 0.12	1.29 ± 0.28	1.99 ± 0.09
**GIV 08**	n.m.	18.6 ± 0.6	23.6 ± 1.4	370.02 ± 72.75	593.05 ± 111.51	550.65 ± 25.75	41.76 ± 0.86	13.94 ± 0.06	6.15 ± 0.01	0.69 ± 0.16	0.76 ± 0.49	2.44 ± 0.33
**MN 08**	n.m.	14.4 ± 0.7	19.3 ± 2.7	68.97 ± 16.05	516.17 ± 35.28	824.54 ± 59.35	35.50 ± 0.49	32.84 ± 0.19	12.34 ± 0.03	0.16 ± 0.03	0.82 ± 0.30	1.14 ± 0.15
**PM 08**	n.m.	17.4 ± 1.2	18.6 ± 0.3	148.19 ± 12.53	179.44 ± 26.06	264.56 ± 30.85	37.86 ± 0.76	12.02 ± 0.11	5.50 ± 0.02	0.69 ± 0.13	2.39 ± 0.37	2.66 ± 0.53
**PSP 08**	n.m.	14.6 ± 0.4	17.8 ± 0.6	108.76 ± 29.95	235.44 ± 50.98	355.39 ± 73.63	40.80 ± 0.26	21.17 ± 0.10	7.75 ± 0.04	0.46 ± 0.14	0.97 ± 0.31	1.76 ± 0.26
**VM 08**	n.m.	15.0 ± 0.8	21.0 ± 1.2	150.45 ± 94.45	308.92 ± 29.69	609.41 ± 32.95	35.82 ± 0.47	20.85 ± 0.11	8.25 ± 0.06	0.57 ± 0.15	1.68 ± 0.34	2.15 ± 0.33

n.m. = not measured

Table S3

Table S3. Maturation parameters of samples used for microarray analysis sorted by year of harvesting. Values represent mean ± standard deviation of three biological replicates. Total acidity is expressed in g/l of tartaric acid. For metabolic parameters, values are expressed as mean peak area ± standard deviation of three biological replicates.
