# Supplementary material for: The plasticity of the grapevine berry transcriptome
Source: Genome Biol. 2013 Jun 7;14(6):r54. doi: 10.1186/gb-2013-14-6-r54 (PMC3706941; doi:10.1186/gb-2013-14-6-r54)
Supplement: Additional File 5 — Figure S1. Cluster dendrogram of (a) the second developmental stage and (b) the third developmental stage datasets using the average expression value of the three biological replicates. The Pearson's correlation values were converted into distance coefficients to define the height of the dendrograms. Blue, green, and red indicate samples harvested in 2006, in 2008, and in 2007, respectively. [file gb-2013-14-6-r54-S5.PDF]

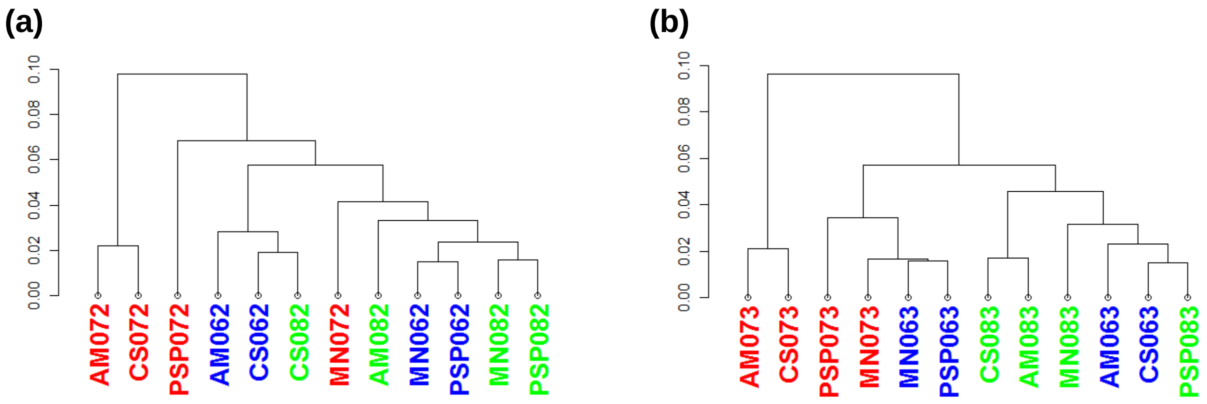

Figure S1

Figure S1. Cluster dendrogram of **(a)** the second developmental stage and **(b)** the third developmental stage datasets using the average expression value of the three biological replicates. The Pearson's correlation values were converted into distance coefficients to define the height of the dendrograms. Blue, green and red indicate samples harvested in 2006, in 2008 and in 2007, respectively.
