# Supplementary material for: The plasticity of the grapevine berry transcriptome
Source: Genome Biol. 2013 Jun 7;14(6):r54. doi: 10.1186/gb-2013-14-6-r54 (PMC3706941; doi:10.1186/gb-2013-14-6-r54)
Supplement: Additional File 7 — Figure S2. Differential accumulation of metabolites between the 2006/2008 and 2007 vintages. Values were calculated as mean peak area ± standard deviation of three biological replicates and are expressed as fold-change of vintages 2006 to 2008 compared to 2007. [file gb-2013-14-6-r54-S7.PDF]

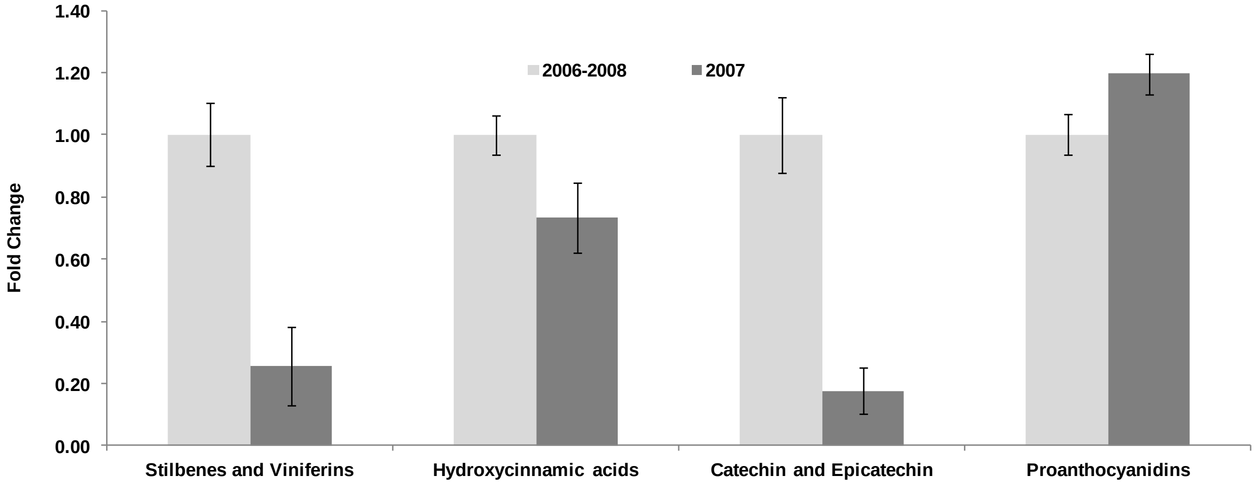

Figure S2

Figure S2. Differential accumulation of metabolites between the 2006/2008 and 2007 vintages. Values were calculated as mean peak area ± standard deviation of three biological replicates and are expressed as fold-change of vintages 2006-2008 compared to 2007.
