# Supplementary material for: The plasticity of the grapevine berry transcriptome
Source: Genome Biol. 2013 Jun 7;14(6):r54. doi: 10.1186/gb-2013-14-6-r54 (PMC3706941; doi:10.1186/gb-2013-14-6-r54)
Supplement: Additional File 8 — Figure S3. Plastic and vintage-specific transcripts. Kruskal-Wallis non-parametric variance analysis was carried out three times (P <0.05, four groups) on each vintage-specific dataset to obtain differentially-modulated genes among the four vineyards studied in each year. The Venn diagram was constructed using Venn [84] and redrawn. [file gb-2013-14-6-r54-S8.PDF]

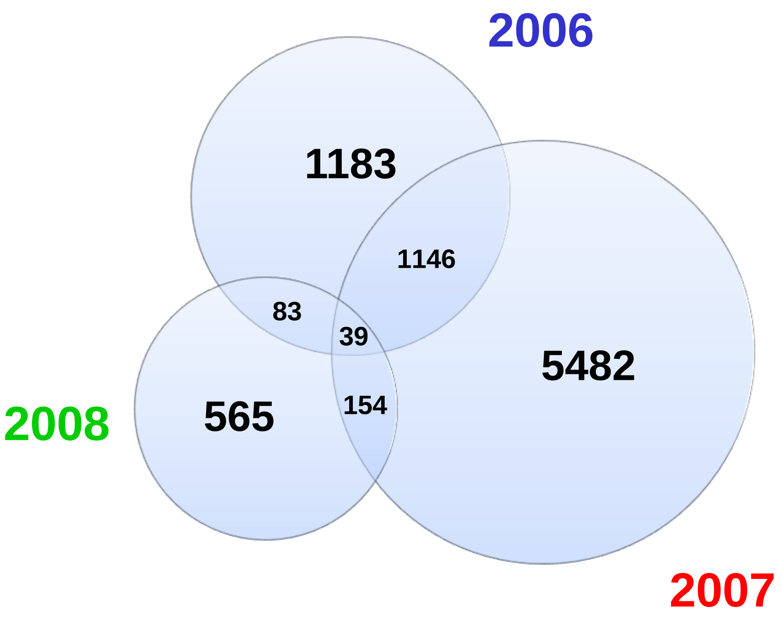


**Figure S3**

**Figure S3.** Plastic and vintage-specific transcripts. Kruskal-Wallis non-parametric variance analysis was carried out three times ($p < 0.05$, four groups) on each vintage-specific dataset to obtain differentially-modulated genes among the four vineyards studied in each year. The Venn diagram was constructed using Venn (http://bioinformatics.psb.ugent.be/webtools/Venn/) and redrawn.
