# Supplementary material for: The plasticity of the grapevine berry transcriptome
Source: Genome Biol. 2013 Jun 7;14(6):r54. doi: 10.1186/gb-2013-14-6-r54 (PMC3706941; doi:10.1186/gb-2013-14-6-r54)
Supplement: Additional File 9 — Figure S4. k-means clustering of fluorescence log2 intensities. Increasing values of k were used until only one cluster displayed bimodal distribution (k = 10) with a low expression level mean value. [file gb-2013-14-6-r54-S9.PDF]

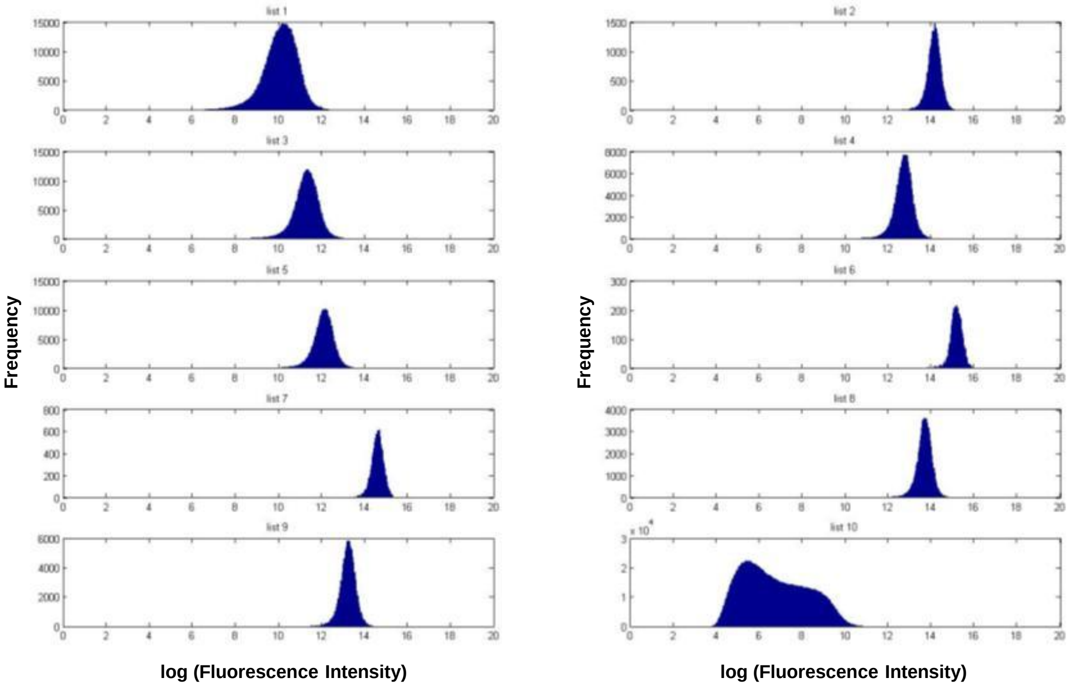

Figure S4

Figure S4. k-means clustering of fluorescence log_2 intensities. Increasing values of k were used until only one cluster displayed bimodal distribution (k = 10) with a low expression level mean value.
