# Supplementary material for: The plasticity of the grapevine berry transcriptome
Source: Genome Biol. 2013 Jun 7;14(6):r54. doi: 10.1186/gb-2013-14-6-r54 (PMC3706941; doi:10.1186/gb-2013-14-6-r54)
Supplement: Additional File 12 — Figure S5. Enriched GO terms for the 1,478 plastic genes listed in Dataset S4. The network graphs show BiNGO visualizations of the overrepresented GO terms. Categories in GoSlimPlants [79] were used to simplify this analysis. Non-colored nodes are not over-represented, but they may be the parents of overrepresented terms. Node size is positively correlated with the number of genes belonging to the category. Colored nodes represent GO terms that are significantly over-represented (P value <0.1), with the shade indicating significance as shown in the color bar. [file gb-2013-14-6-r54-S12.PDF]

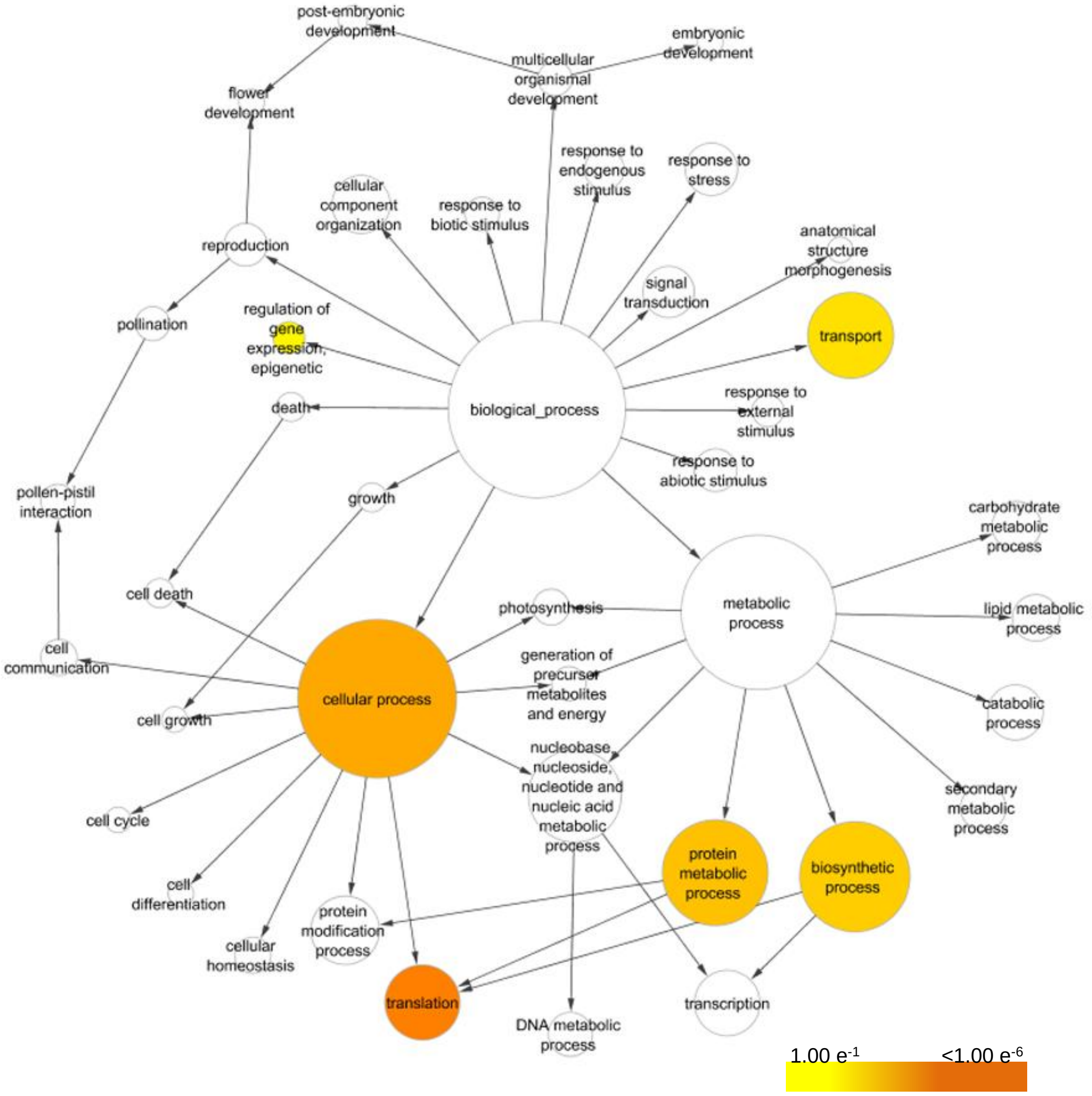


# Figure S5

**Figure S5.** Enriched GO terms for the 1478 plastic genes listed in Dataset S4. The network graphs show BiNGO visualizations of the overrepresented GO terms. Categories in GoSlimPlants [71] were used to simplify this analysis. Non-colored nodes are not overrepresented, but they may be the parents of overrepresented terms. Node size is positively correlated with the number of genes belonging to the category. Colored nodes represent GO terms that are significantly overrepresented (p value < 0.1), with the shade indicating significance as shown in the color bar.
