# Supplementary material for: The plasticity of the grapevine berry transcriptome
Source: Genome Biol. 2013 Jun 7;14(6):r54. doi: 10.1186/gb-2013-14-6-r54 (PMC3706941; doi:10.1186/gb-2013-14-6-r54)
Supplement: Additional File 13 — Figure S6. Validation of O2PLS-DA model. The three-latent-component O2PLS-DA model in Figure 3E was partially cross-validated and a permutation test (100 permutations) was used to highlight putative overfitting. [file gb-2013-14-6-r54-S13.PDF]

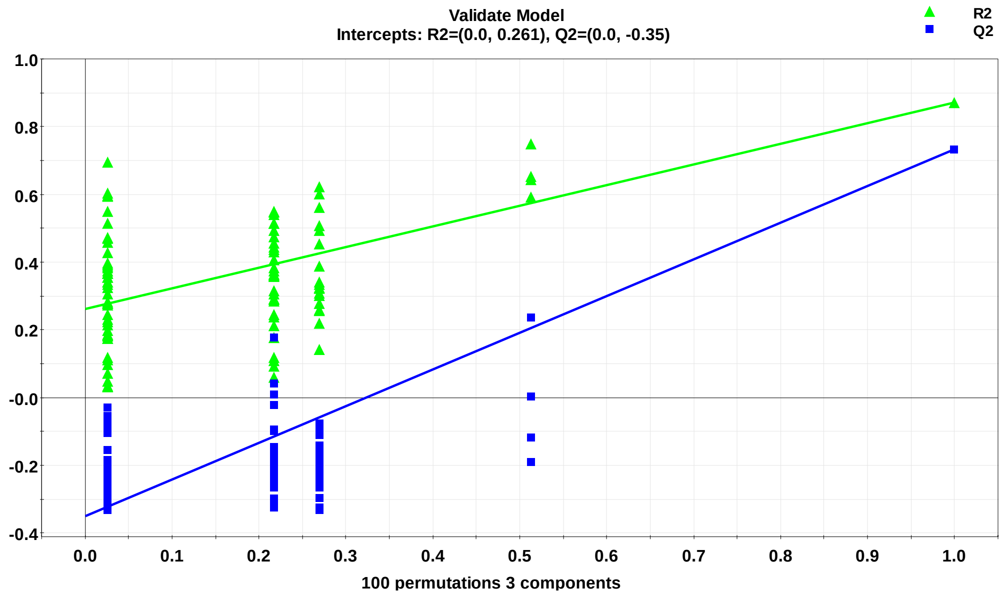

Figure S6

Figure S6. Validation of O2PLS-DA model. The three-latent-component O2PLS-DA model in Figure 3E was partially cross-validated and a permutation test (100 permutations) was used to highlight putative overfitting.
