# Supplementary material for: The plasticity of the grapevine berry transcriptome
Source: Genome Biol. 2013 Jun 7;14(6):r54. doi: 10.1186/gb-2013-14-6-r54 (PMC3706941; doi:10.1186/gb-2013-14-6-r54)
Supplement: Additional File 19 — Figure S8. Differentially modulated genes at harvesting. (a) Cluster dendrogram of the third developmental stage dataset using the average expression value of the three biological replicates. Pearson's correlation values were converted into distance coefficients to define the height of the dendrogram. Different colors indicate the disparity in the degree of ripening as analyzed in (c). (b) Differentially-expressed genes between the two groups of vineyards highlighted in (a). A t-test (α = 0.05) was performed between the two groups of vineyards, and a k-means analysis was computed using Pearson's distance to generate the line plots. (c) Functional category distribution of the differentially-modulated genes between the two groups of vineyards during harvesting. Transcripts were grouped into the 18 most represented functional categories, based on Plant GO Slim classification of biological processes. Sample VM083, GIV083, CC083, PM083, AM083, and FA083 category distribution is depicted in purple, while sample CS083, PSP083, BA083, BM083, and MN083 category distribution is depicted in light green. (d) Total acidity, expressed in g/L of tartaric acid and °Brix/total acidity of samples from the third developmental stage. Values represent mean ± standard deviation of three biological replicates. Different colors indicate the disparity in the degree of ripening as shown in (a) and in (c). [file gb-2013-14-6-r54-S19.PDF]

(a)

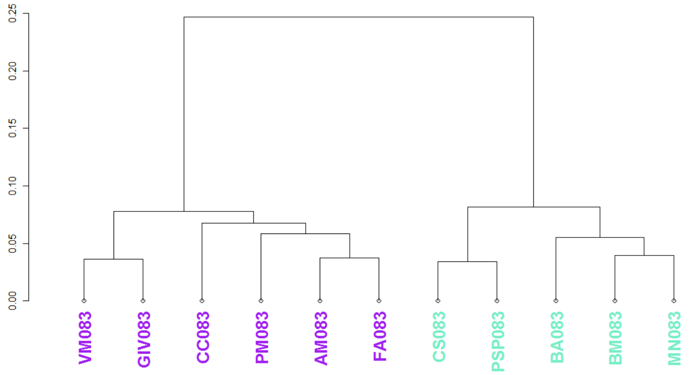

(d)

	Total Acidity	°BRIX/Total Acidity
BM 08 3	10.46 ± 0.01	1.93 ± 1.60
BA 08 3	8.53 ± 0.01	2.09 ± 1.00
MN 08 3	8.44 ± 0.02	2.29 ± 2.70
AM 08 3	6.03 ± 0.05	3.30 ± 0.30
CS 08 3	5.82 ± 0.04	3.38 ± 2.00
PSP 08 3	5.09 ± 0.01	3.50 ± 0.60
FA 08 3	4.53 ± 0.01	3.91 ± 0.30
CC 08 3	4.88 ± 0.05	4.16 ± 1.80
VM 08 3	4.74 ± 0.03	4.43 ± 1.20
PM 08 3	4.15 ± 0.01	4.48 ± 0.30
GIV 08 3	4.30 ± 0.06	5.49 ± 1.40

(b)

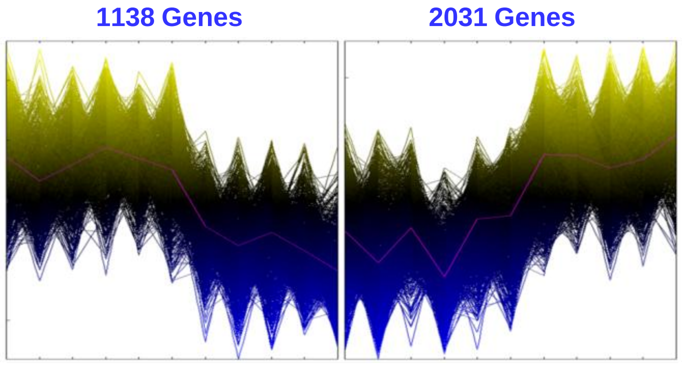

(c)

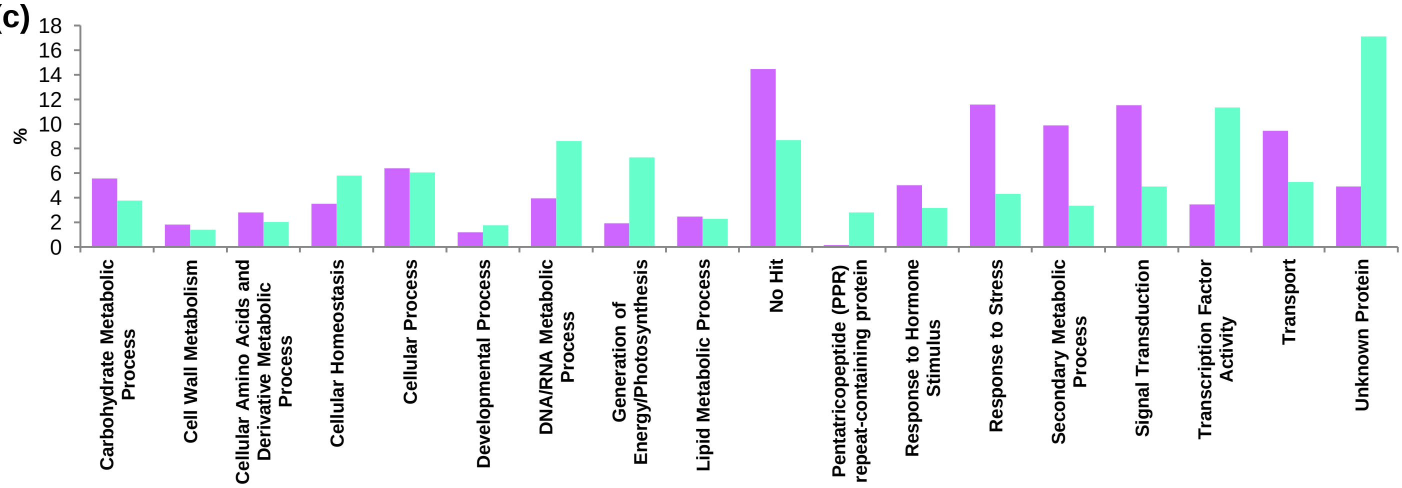

Figure S8

Figure S8. Differentially modulated genes at harvesting.
(a) Cluster dendrogram of the third developmental stage dataset using the average expression value of the three biological replicates. Pearson's correlation values were converted into distance coefficients to define the height of the dendrogram. Different colors indicate the disparity in the degree of ripening as analyzed in (c).
(b) Differentially-expressed genes between the two groups of vineyards highlighted in (a). A t-test ($\alpha = 0.05$) was performed between the two groups of vineyards, and a k-means analysis was computed using Pearson's distance to generate the line plots.
(c) Functional category distribution of the differentially-modulated genes between the two groups of vineyards during harvesting. Transcripts were grouped into the 18 most represented functional categories, based on Plant GO Slim classification of biological processes. Sample VM083, GIV083, CC083, PM083, AM083 and FA083 category distribution is depicted in purple, while sample CS083, PSP083, BA083, BM083 and MN083 category distribution is depicted in light green.
(d) Total acidity, expressed in g/l of tartaric acid and °Brix/total acidity of samples from the third developmental stage. Values represent mean ± standard deviation of three biological replicates. Different colors indicate the disparity in the degree of ripening as shown in (a) and in (c).
