# Supplementary material for: The plasticity of the grapevine berry transcriptome
Source: Genome Biol. 2013 Jun 7;14(6):r54. doi: 10.1186/gb-2013-14-6-r54 (PMC3706941; doi:10.1186/gb-2013-14-6-r54)
Supplement: Additional File 21 — Figure S9. Non-plastic genes. Stage-specific datasets were analyzed by SAM multiclass analysis and one-way ANOVA (11 groups). Transcripts not shown to be significant in either analysis (that is, not differentially modulated) were tested for stage-specificity. The Venn diagram was calculated using Venn [84] and redrawn. [file gb-2013-14-6-r54-S21.PDF]

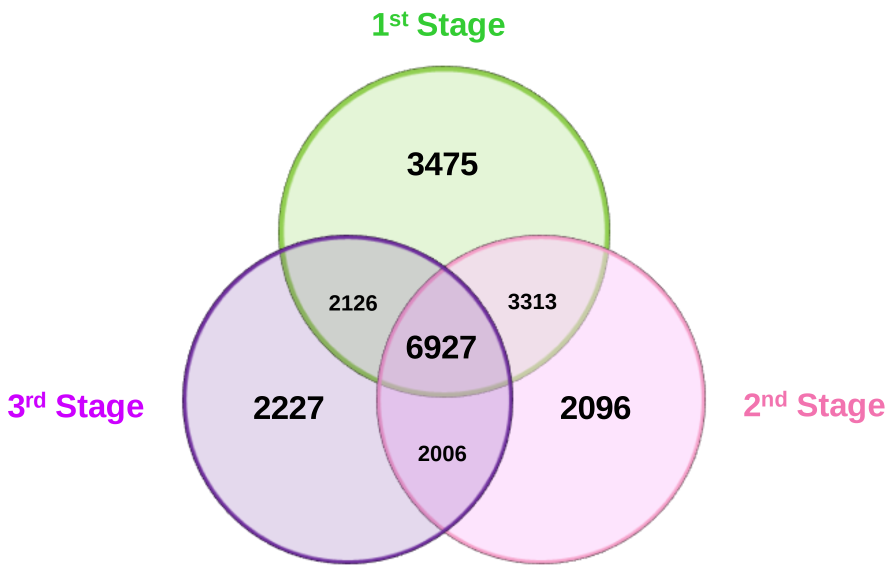

Figure S9

Figure S9. Non-plastic genes. Stage-specific datasets were analyzed by SAM multiclass analysis and one-way ANOVA (11 groups). Transcripts not shown to be significant in either analysis (i.e. not differentially modulated) were tested for stage-specificity. The Venn diagram was calculated using Venn (http://bioinformatics.psb.ugent.be/webtools/Venn/) and redrawn.
