# Supplementary material for: The plasticity of the grapevine berry transcriptome
Source: Genome Biol. 2013 Jun 7;14(6):r54. doi: 10.1186/gb-2013-14-6-r54 (PMC3706941; doi:10.1186/gb-2013-14-6-r54)
Supplement: Additional File 15 — Figure S7. Hierarchical clustering analysis of environment-specific differentially expressed genes in (a) vineyards using parral or Guyot replacement cane trelling systems and (b) vineyards located in one of the three macro-areas we investigated. Pearson's correlation distance was used as the metric. The heat map of transcriptional profiles was generated with TMeV 4.8 using the average expression value of the three biological replicates per each developmental stage. [file gb-2013-14-6-r54-S15.PDF]

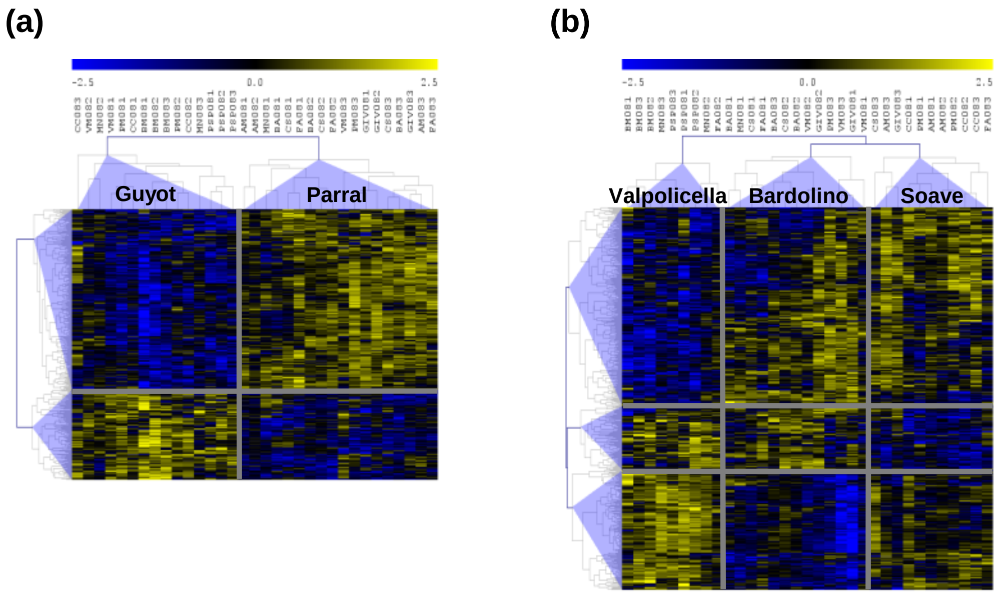

Figure S7

Figure S7. Hierarchical clustering analysis of environment-specific differentially expressed genes in **(a)** vineyards using parral or Guyot replacement cane trelling systems and **(b)** vineyards located in one of the three macro-areas we investigated. Pearson's correlation distance was used as the metric. The heat map of transcriptional profiles was generated with TMeV 4.8 using the average expression value of the three biological replicates per each developmental stage.
