# Supplementary material for: The plasticity of the grapevine berry transcriptome
Source: Genome Biol. 2013 Jun 7;14(6):r54. doi: 10.1186/gb-2013-14-6-r54 (PMC3706941; doi:10.1186/gb-2013-14-6-r54)
Supplement: Additional File 23 — Figure S10. Expression profile of five non-plastic constitutive genes in the whole grapevine expression atlas [33]. [file gb-2013-14-6-r54-S23.PDF]

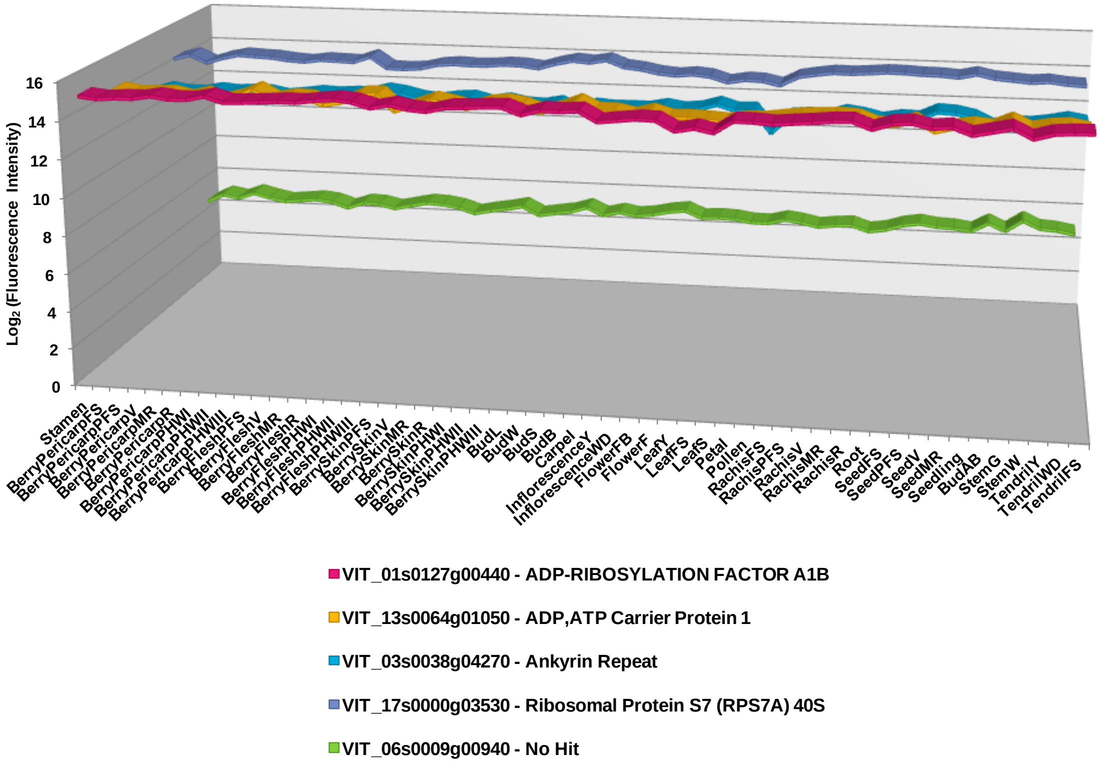

Figure S10

Figure S10. Expression profile of five non-plastic constitutive genes in the whole grapevine expression atlas [31].
